# *Lactobacillus rhamnosus*-derived extracellular vesicles influence calcium deposition in a model of breast cancer intraductal calcium stress

**DOI:** 10.1016/j.isci.2025.112538

**Published:** 2025-04-28

**Authors:** Ngoc Vuong, Melany Alomia, Ahana Byne, Purva Gade, Thomas Raymond Philipson, Rayan Ibrahim Alhammad, Cade J. Skislak, Intisar Alruwaili, Fahad M. Alsaab, Weidong Zhou, Marissa Howard, Andrea Brothers, Amanda Haymond Still, Robyn P. Araujo, Monique Van Hoek, Barbara Birkaya, Virginia Espina, Richard A. Hoefer, Lance Liotta, Alessandra Luchini

**Affiliations:** 1 Center for Applied Proteomics and Molecular Medicine, George Mason University, 10920 George Mason Circle, MSN 1A9, Manassas, VA 20110, USA; 2 School of Systems Biology, George Mason University, 10920 George Mason Circle, MSN 1A9, Manassas, VA 20110, USA; 3 College of Applied Medical Sciences, King Saud Bin Abdulaziz University for Health Sciences, Al Ahsa, Saudi Arabia; 4 College Dean’s Office, American University, 4400 Massachusetts Avenue, Washington, DC 20016, USA; 5 The University of Melbourne, Grattan Street, Parkville, VIC 3010, Australia; 6 Dorothy G. Hoefer Comprehensive Breast Center, 11803 Jefferson Avenue, Suite 130, Newport News, VA 23606, USA; 7 Clinical Laboratory Department, College of Applied Medical Science, King Saud bin Abdulaziz University for Health Sciences, Al Ahsa, Saudi Arabia; 8 King Abdullah International Medical Research Center, Al Ahsa, Saudi Arabia

**Keywords:** Molecular biology, Microbiology, Cell biology, Cancer

## Abstract

Extracellular calcium export by the breast ductal epithelium is crucial during lactation and plays a significant role in breast cancer progression. Intraductal calcium deposition is a hallmark of aggressive premalignant lesions. This study tested the hypothesis that microbiome-derived extracellular vesicles (EVs) influence calcium modulation in premalignant breast cancer lesions. Based on the analysis of plasma, serum, saliva, and tissue collected from breast cancer patients and controls (*N* = 150), *Lactobacillus rhamnosus* (*Lr*) was chosen as the model microbiota. In a BT-474 human breast cancer cell line monolayer culture under acute calcium stress, *Lr* EVs enhanced intracellular calcium intake. In a BT474 3D spheroid model under chronic calcium stress, *Lr* EVs increased extracellular calcium deposition and mRNA expression of calcium export channel plasma membrane calcium-transporting ATPase 2 (PMCA2) and stromal interaction molecule 1 (STIM1) in a dose-dependent manner. We propose that *Lr* EVs influence calcium regulation and mineral deposition, thereby affecting premalignant breast cancer progression.

## Highlights

• *Lr* EV markers are found in blood and tissue biopsies of breast cancer patients
• *Lr* EVs increase calcium intake in BT-474 cells under acute calcium stress
• Lr EVs increase microcalcifications and PMCA2/STIM1 mRNA in BT-474 spheroids
• *Lr* EVs influence calcium regulation, thus affecting breast cancer progression

## Graphical abstract

**Figure:**
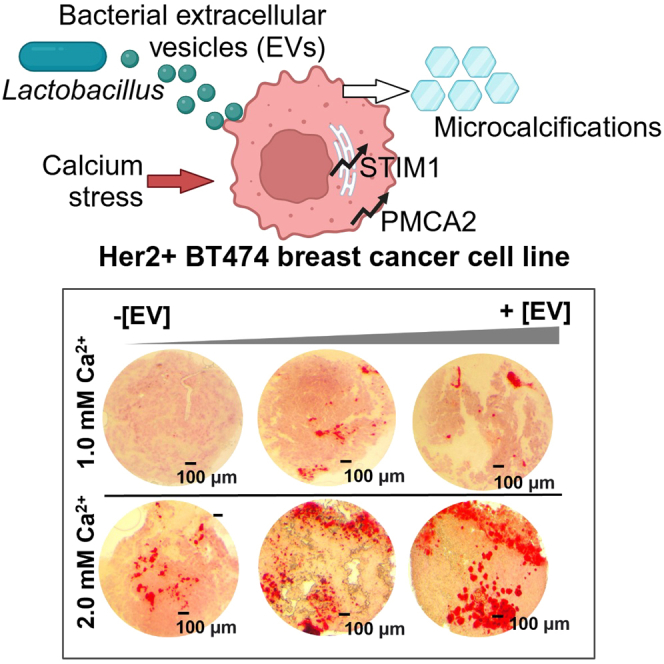

## Introduction

Recent studies have focused on the human microbiome as a potential risk factor for breast cancer etiology and progression.^1^^,^^2^^,^^3^^,^^4^ Breast tissue is not a sterile environment, but is populated by diverse microbiota, including predominant phyla such as *Firmicutes*, *Proteobacteria*, *Actinobacteria*, and *Bacteroidetes*.^4^^,^^5^ The microbiota in healthy tissues has been reported to include a larger number of genera and species, whereas breast cancer tissues show a decrease in both abundance and diversity.^3^^,^^4^^,^^6^ Despite offering exciting correlations, these studies have provided very few insights into the mechanistic and functional roles of the microbiome in carcinogenesis. Consequently, there is an unmet need to develop microbiome analysis methods that provide functional information regarding products derived from microbes that mechanistically influence the local or systemic host microenvironment to promote or suppress cancer.^7^^,^^8^ Recently, there has been growing interest in the importance of microbial-derived extracellular vesicles (EVs) as intercellular and interkingdom communication agents that connect microbial communities and modulate host cell responses.^9^ Bacterial EVs contain diverse bioactive molecules, including proteins, lipids, DNA, RNA, and metabolites, that can be shuttled from the bacteria to the host.^10^

Extracellular calcium export via calcium effluxes/channels in the breast epithelium is essential for milk production. Intraductal calcium mineral deposition, usually most prominent within large hypoxic high-grade intraductal carcinoma lesions, is a common feature of pre-malignant lesion pathology.^11^^,^^12^ Intraductal calcification was significantly increased in breast cancers with HER2 overexpression.^13^ Although the functional roles and physiology of breast intraductal calcium deposition remain unknown, calcium handling plays a well-established role in breast cancer progression,^11^^,^^14^ which has never been experimentally connected to the microbiome. Intracellular calcium modulation is tightly regulated, because too much or too little calcium is cytotoxic. This strict calcium homeostasis is regulated by various calcium channels and sensors, including plasma membrane calcium-transporting ATPase 2 (PMCA2), stromal interaction molecule 1 (STIM1), and calcium channel protein 1 (ORAI-1). We and others^15^^,^^16^ have previously shown that calcium export channels, such as PMCA2, play an important role in supporting breast cancer cell survival and premalignant progression in the face of high extracellular calcium stress.

We hypothesized that the intraductal microbiome influences calcium handling and mineral deposition in breast cancer cells by engaging calcium pumps/channels. In this study, we investigated the microbiome circulating proteome in a clinical study of 150 women who underwent a follow-up biopsy after receiving a suspicious mammogram. *Lactobacillus rhamnosus* (*Lr*), of the *Lactobacillus* genus, which was more represented in breast cancer cases than controls, was chosen as a model commensal bacterium. We created an *in vitro* model to evaluate the previously unknown role of human microbiota in influencing calcium regulation and deposition in breast cancer. We tested the influence of microbiome EVs on intracellular calcium levels using a BT-474 human HER2-positive breast cancer cell line in a monolayer culture model exposed to short-term calcium stress. We developed a BT-474 3D spheroid model that approximates the breast intraductal hypoxic gradient, with associated metabolic acidosis and increased intracellular calcium levels. We tested whether microbiome EVs could influence extracellular calcium deposition in the growing spheroids. We recorded the relationship between supplemented calcium in the culture medium and the deposition of calcium salts within the growing spheroids, with and without bacterial EV treatment, at a series of doses. To explore potential molecular mechanisms related to calcium transport, we evaluated the transcription of calcium channels/pumps in breast cancer spheroids exposed to different environmental calcium levels and bacterial EV doses. We investigated the microbiome derived molecular deposition in human ductal carcinoma *in situ* (DCIS) tissues. The overall goal was to create an experimental model and workflow from patient microbiome proteomics to reveal novel measurable molecular mechanisms by which the microbiome can influence various aspects of breast cancer pathogenesis.

## Results

### Patient cohort

**Figure 1:**
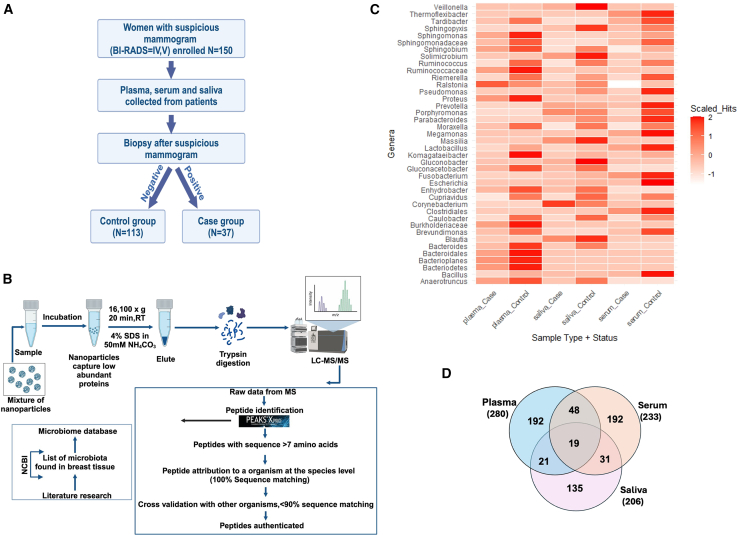
Experimental pipeline and results of microbiome proteomic analysis (A) Samples (plasma, serum and saliva) were collected from 150 patients who received a suspicious mammogram, and breast cancer was investigated by follow-up biopsy. *N* = 37 patients had positive biopsy and *N* = 113 had negative biopsy. Samples were obtained after informed consent and following the procedures and guidelines of the Sentara Dorothy G. Hoefer Comprehensive Breast Center at the time of biopsy. (B) Overview of the mass spectrometry analysis workflow for plasma, serum, and saliva samples. Low-molecular-weight, low-abundance proteins were captured using a mixture of nanoparticles and eluted for downstream mass spectrometry analysis. Created in https://BioRender.com. (C) Heatmap showing differences in microbiota genus abundance between breast cancer patients and controls in three body fluids (plasma, serum, and saliva). The data were measured using a *Z* score scale. (D) Venn diagram of microbiome genera identified in plasma, serum and saliva via mass spectrometry.

Our study included 150 women who had suspicious mammogram results and were subjected to follow-up biopsy within one year (Figure 1A). Clinical and demographic information for breast cancer patients, including age, family history of breast cancer, tumor type, HER2 status, and BI-RADS score, are reported in Tables 1 and 2. The median subject age was 54.3 years; 64% of the women were postmenopausal and 74% were scored with a Breast Imaging Reporting and Data System (BI-RADS) rating of 4. All patients were diagnosed with a BI-RADS score of IV or V and underwent a follow-up breast biopsy; 75.3% of the pathological diagnoses were negative for breast cancer. No association was found between family history of breast cancer and breast cancer diagnosis (Chi square X^2^ = 1.44, *p* = 0.23). Similarly, no relationship was found between menopausal status and breast cancer (Chi square X^2^ = 0.18, *p* = 0.66).

**Table 1:** The study included 150 women with suspicious mammograms, followed by tissue biopsy

Total	150
**Age**	

<50 years	60
≥50 years	90

**Menopause status**	

Pre Post Unknown	50
	96
	4

**BI-RADS scores**	

IV	111
V	39

**Family history of breast cancer**	

Yes	65
No	84
Unknown	1

**ER**	

Positive	27
Negative	6
Unknown	4

**HER-2**	

Positive	11
Negative	14
Unknown	12

**Biopsy result**	

Positive	37
Negative	113

The table reports demographic and clinical information of the patient set.

**Table 2:** Race and ethnicity distribution of women undergoing mammographic screening at Sentara Dorothy G. Hoefer Comprehensive Breast Center during the study period

Race/ethnicity	2022	2023
0 - Not Specified	0.21%	0.16%
1 - American Indian	0.41%	0.41%
2 - Asian	3.15%	3.53%
3 - Black	47.15%	47.83%
5 - White	47.86%	46.83%
6 - Other-Unknown	0.57%	0.52%
7 - Pacific Islander	0.40%	0.44%
8 - Patient Refused	0.25%	0.28%

### *Lactobacillus rhamnosus* is associated with breast cancer cases in a microbiome proteome analysis of plasma, serum, and saliva

Follow-up biopsy results were used to divide the study participants into two classes: breast cancer patients (*N* = 37) and negative controls (*N* = 113). Plasma, serum, and saliva samples were collected from 150 women at the time of biopsy and analyzed using mass spectrometry proteomic analysis (Figure 1B). Mass spectrometry analysis yielded 515, 552, and 301 microbiome-authenticated peptides in serum, plasma, and saliva, respectively, for a total of 1066 peptides (Tables S1, S2, and S3). Peptide sequences were authenticated to have 100% homology with a microbiota at the genus or species level and less than 90% homology with any other organism in the NCBI non-redundant database.

Identified microbiome derived peptides (Tables S1, S2, and S3; Figures S1–S3) were related to nucleotide and amino acid metabolism (aminotransferases, phosphoribosyltransferases, and proteins involved in biosynthesis and degradation of nucleic acids and amino acids), transport (ion, carbohydrate, protein, and ABC transporters), carbohydrate and energy metabolism (glycolysis proteins, ATP synthase), protein folding/chaperones (heat shock proteins, DnaK family proteins), protein degradation (proteases, peptidases, metallopeptidases), DNA/RNA metabolism (DNA synthesis, replication, degradation, and repair proteins; RNA synthesis, transcription, modification, processing, and degradation proteins), signal transduction (kinases, phosphatases, GTP-binding proteins), protein translation and modification (aminoacyl tRNA synthetases, elongation factors, components of small subunit and large subunit ribosomal proteins, glycosyltransferases), lipid metabolism (proteins involved in lipid transport, biosynthesis, and degradation), surface/secreted antigens, cell wall remodeling (surface antigen, outer membrane proteins, peptidoglycan biosynthesis and degradation enzymes), motility (pilus proteins), redox homeostasis (oxidoreductases and other proteins that maintain the redox environment of the cell).

At the peptide level, the microbiome peptidome exhibited greater diversity (Chi square X^2^ = 47.353, *p* = 5.93e-12; Chi square X^2^ = 65.253, *p* = 3.909e-13; Chi square X^2^ = 68.291, *p* = 2.866e-10 in plasma, serum, and saliva, respectively) and abundance (Wilcoxon rank-sum test *p* = 0.03434, *p* = 9.807e-05, *p* = 0.06434 in plasma, serum, and saliva, respectively) in controls versus cases.

The authenticated peptides belonged to five phyla (Actinobacteria, Fusobacteria, Bacteroidetes, Proteobacteria, and Firmicutes). At the genus level, 32 genera were detected in the plasma, serum and saliva (Figure 1C), with *Bacillus*, *Bacteroides*, and *Lactobacillus* accounting for approximately 80% of the bacterial counts in each biofluid set. Bacterial genera represented by at least two unique peptides tended to be higher in the controls (plasma: 20 and 12 genera were represented by 1773 and 456 peptide hits in controls and cases, respectively, Wilcoxon rank-sum test *p* = 0.315; serum controls had 4046 peptide hits and 19 genera, cases had 1021 peptide hits and 13 genera, Wilcoxon rank-sum test *p* = 0.3498; Figure 1C, saliva control peptide hits = 1233, 22 genera, saliva case peptide hits = 801, 14 genera, Wilcoxon rank-sum test *p* = 0.03875; Figure 1C). Nevertheless, exceptions were observed, including *Ruminococcus* in plasma with peptide hit numbers in cases being 1.57 times higher than in controls, and *Prevotella* in serum or *Sphingopyxis* in saliva with 1.75 times and 2.33 times higher peptide hit numbers in cases than in controls, respectively. Three genera were found in only one body fluid type: *Thermoflexibacter* and *Veilonella* in the serum and *Massilia* in the plasma. In addition, though with low peptide abundances, *Blautia*, *Enhydrobacter*, and *Sphingopyxis* were only detected in the saliva. When correlating the microbiome with clinical variables, we noted that serum *Lactobacillus* peptide hits were 1.3 times more abundant in HER2 positive than HER2 negative cases (Figure S4).

At the species level, serum yielded the largest number of microbial species (*N* = 233), followed by plasma (*N* = 280) and saliva (*N* = 206, Figure 1D). Plasma and serum also shared a greater similarity of identified organisms at the species level (17.4%) than the overlap observed in plasma and saliva (15.2%), and in serum and saliva (12.2%).

**Figure 2:**
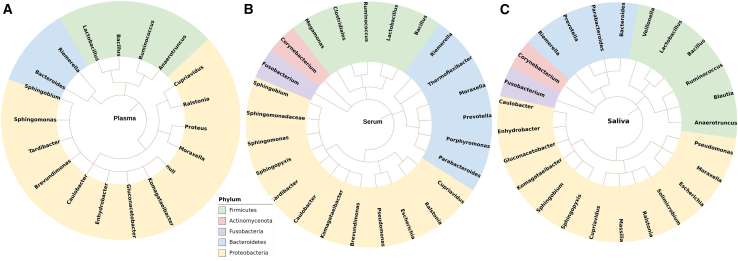
Phylogenetic trees of serum, plasma and saliva microbiome proteomes Phylogenetic trees for plasma (A), serum (B) and saliva (C) microbiome proteomes are shown at the genus level. The corresponding species are presented in Data S1, S2, and S3. This figure was obtained using the Interactive Tree of Life (iTOL) software and taxonomy IDs from NCBI.

To visualize the circulating peptidome of the microbiome, we generated three circular taxonomic maps that showed all bacterial species found in the plasma, serum, and saliva. The bacterial groups commonly found in previous breast microbiome studies were confirmed in our datasets (Figure 2; Data S1, S2, and S3), including *Bacillus*, *Lactobacillus*, *Bacteroides*, *Corynebacterium*, *Escherichia*, *Fusobacterium*, *Gluconacetobacter*, *Prevotella*, *Pseudomonas*, *Ruminococcus*, and *Sphingomonas*.^5^^,^^17^ As expected based on published literature,^18^ controls had a higher number of species than cases, although saliva species failed to reach statistical significance (151 and 38 species identified in plasma, Chi square X^2^ = 29.473, *p* = 5.671e-08; 145 and 64 species found in serum, Chi square X^2^ = 15.338, *p* = 8.988e-05; 90 and 44 species found in saliva, Chi square X^2^ = 1.6647e-27, *p* = 1). However, some bacterial species showed opposite trends and were more abundant in cases than in controls. Plasma and serum *L. rhamnosus* (*Lr*) peptide hits were more abundant in cases than in controls (linear model case group coefficient = 41, standard error = 15, adjusted *p* = 0.043) and in patients with a BI-RADS score of 5 than 4 (linear model BI-RADS 5 coefficient = 56, standard error = 6, adjusted *p* = 0.0005). Based on these findings, we chose *Lr* as a model Gram-positive *Lactobacillus* microbiota to investigate its influence on calcium regulation in breast cancer spheroids.

### Morphological and proteomic characterization of *Lactobacillus rhamnosus*-derived EVs

**Figure 3:**
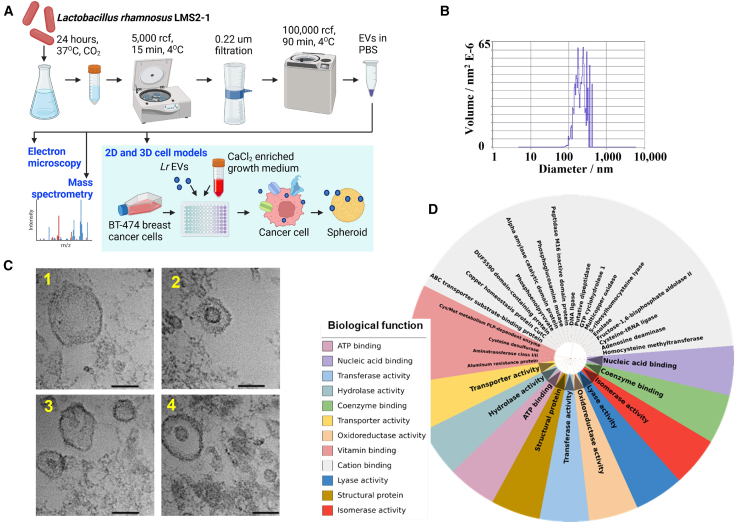
Morphological and proteomic characterization of *L*. *rhamnosus LMS2-1*-derived EVs (A) Overview of the 3D spheroid-bacterial EVs experiment. *L*. *rhamnosus* bacteria were cultured overnight at 37°C and EVs were isolated by ultracentrifugation; the EVs were characterized in terms of morphology, size, and protein content. BT-474 breast cancer spheroids were maintained for two weeks using culture medium supplemented with CaCl_2_ at a total calcium concentration of up to 2.0 mM and with *Lactobacillus rhamnosus LMS2-1*-derived EVs. Created in https://BioRender.com. (B) Representative particle size analysis of *L*. *rhamnosus* EVs (particles/mL). (C) Representative transmission electron microscopy (TEM) images of *L*. *rhamnosus* EVs. The EVs displayed diameters ranging from ∼60 to 200 nm, spherical morphology, and differentially electron dense cores. The scale bar is 100 nm. (D) Mass spectrometry analysis of *L*. *rhamnosus*-EVs yielded 174 bacterial proteins with different biological functions. The image was generated from iTOL (https://itol.embl.de).

Standard bacterial EV collection and purification strategies were applied to the *L. rhamnosus LMS2-1* strain (Figure 3A). EV production increased with bacterial titer, reaching a particle count of 5.3 × 10^10^ per mL after 4.5 h of culture (OD600 = 0.6) and 1.3 × 10^12^ per mL after 24 h of culture (Figures S5 and S6). This result is in line with those of previous studies where the proportions of bacterial cells and bacterium-derived EVs were positively correlated.^19^ The quantity of EVs released from 10^9^ *Lr* CFUs was 3.50 ± 0.54 × 10^11^ particles per mL (50 mL), with a median size of ∼30–150 nm by ZetaView analysis (Figure 3B). The EVs were imaged using transmission electron microscopy (TEM, Figure 3C). TEM images showed a population of membrane-enclosed, spherical vesicles with sizes ranging from ∼60 to 200 nm (*N* = 150 EVs measured). TEM images showed that smaller vesicles (∼60–100 nm) were more electron dense than larger vesicles (∼100–200 nm). *Lr*-derived EVs were analyzed using mass spectrometry to determine their proteomic content (Figure 3D). The total number of unique bacteria-derived proteins was 174. The identified bacterial EV proteins participate in various biological processes, including glycolysis and other metabolic pathways (pentose phosphate and hexosamine biosynthesis), vitamin binding, ATP binding, nucleic acid binding, coenzyme binding, isomerase activity, lyase activity, oxidoreductase and hydrolase activity, transporter and transferase activity, and cation binding. Specifically, proteins involved in metabolic pathways thought to influence calcium signaling and homeostasis, such as glucokinase, phosphofructokinase, and enolase, were identified in bacterial EVs. Proteins associated with bacterial capsular polysaccharide synthesis, cell wall/envelope biosynthesis, phospholipid synthesis, and transpeptidase (i.e., D-alanine-D-alanine ligase and DD-transpeptidase) were also identified (Table 3; Table S4). These proteins are candidate markers for EVs derived from *Lr* LMS2-1 cells. A subset of 27 bacterial EV proteins overlapped with proteins measured in plasma, serum, or saliva (Table 4), including metabolic enzymes such as diphosphomevalonate decarboxylase involved in the mevalonate pathway and structural proteins such as the LPXTG-motif cell wall anchor domain protein.

**Table 3:** List of proteins found in *Lactobacillus rhamnosus* EVs and their biological functions

Protein composition of *Lactobacillus rhamnosus*-EVs	Biological features	Measured in Gram- positive bacteria EVs
β- lactamase, Penicillin-binding protein	Antibiotic resistance	Kim et al.^20^
von Willebrand factor type A domain protein	Coagulation	Kim et al.^20^
LPXTG-motif cell wall anchor domain protein, Efflux ABC transporter	Cell wall anchor	Murase et al.^21^
30S ribosomal subunit, 50S ribosomal subunit	Cytoplasmic proteins	Murase et al.^21^

**Table 4:** List of proteins found in *Lactobacillus rhamnosus*-EVs that were also identified in saliva, serum, and plasma of cancer patients

Uniprot ID	Bacterial EV Protein Description	Body Fluid
C2K146	5-methyltetrahydropteroyltriglutamate--homocysteine methyltransferase	Serum, Plasma
C2K1D8	ABC transporter substrate binding protein	Saliva, plasma
C2JU76	ABC transporter, permease protein	Plasma
C2JX01	Acyltransferase	Serum
C2JXM2	Adenine phosphoribosyltransferase	Saliva
C2JZB8	Adenosine deaminase	Plasma
C2JT25	Aminotransferase, class I/II	Serum, Plasma
C2JTS9	Beta-galactosidase	Serum
C2JV72	Cys/Met metabolism PLP-dependent enzyme	Plasma
C2JWT5	Dihydrolipoyl dehydrogenase	Plasma
C2JWC4	Dipeptidase	Serum
C2JXB8	Diphosphomevalonate decarboxylase	Serum
C2JY30	DNA polymerase I	Plasma
C2JVZ3	DUF5067 domain-containing protein	Plasma
C2JYK8	Energy-coupling factor transporter ATP-binding protein EcfA	Serum
C2JY52	Foldase protein PrsA	Serum
C2JX37	Glycerol-3-phosphate acyltransferase	Plasma
C2JTZ5	KxYKxGKxW signal domain protein	Plasma
C2JUQ2	LPXTG-motif cell wall anchor domain protein	Serum
C2JUH3	Nicotinate phosphoribosyltransferase	Serum
C2JVJ0	Phage portal protein, HK97 family	Saliva, Serum, Plasma
C2JVK1	Phage tail tape measure protein, TP901 family	Saliva, Serum, Plasma
C2JVV3	Phosphoglycerate kinase	Plasma
C2K0E9	PTS system mannose/fructose/sorbose family IID component	Serum
C2JYA5	Pyruvate oxidase	Saliva
C2JV13	RDD family protein	Serum
C2JV50	YhgE/Pip domain protein	Saliva, Serum

These results provide morphological and proteomic characterization of previously uncharacterized *Lr* EVs and support the presence of *Lactobacillus*-derived bioanalytes in peripheral biofluids.

### *Lactobacillus rhamnosus* EV immunoassay characterization

**Figure 4:**
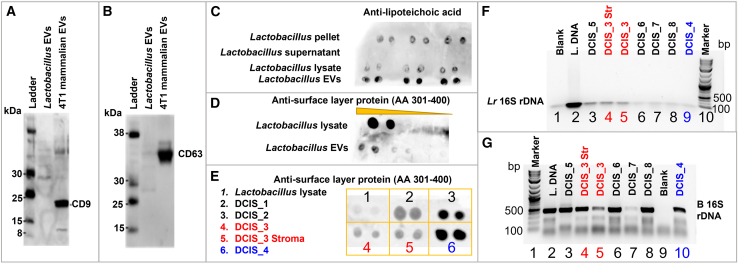
*L*. *rhamnosus* EVs marker identification and detection in ductal carcinoma *in situ* biopsy specimens (A and B) Western blotting showed no evidence of eukaryotic markers (CD63 and CD9) in *L*. *rhamnosus* EVs; EVs derived from 4T1 mouse breast cancer cells were used as positive controls. (C) *L*. *rhamnosus* pellet, lysate, and EVs stained positive for lipoteichoic acid (first, third and fourth lane of the blot, respectively). The second lane of the blot (*Lactobacillus* supernatant) was not positive for lipoteichoic acid. (D) *L*. *rhamnosus* lysate and EVs stained positive for S-layer protein. (E) Lysate from whole slide scraped tissue (sample 2–6) were positive for S-layer protein. DCIS_1 to _4 are tissue slides with high-grade DCIS solid or comedonecrosis lesions. DCIS_3 stroma is a tissue section of non-cancerous tissue. *L*. *rhamnosus* lysate (sample 1) was used as a positive control. (F) PCR amplification and agarose gel separation showed the presence of *L*. *rhamnosus*-specific 16S rDNA in DCIS_3 to DCIS_8 samples. DCIS_5 is a laser-capture microdissected duct tissue and supports the presence of bacterial DNA in the intraductal environment. DCIS_3 to_4 and DCIS_6 to _8 represent whole tissue extractions. (G) PCR amplification and agarose gel separation show the presence of bacterial 16S rDNA in DCIS_3 to DCIS_8 samples. DCIS_5 is laser capture microdissected ducts and supports the presence of bacterial DNA in the intraductal environment. DCIS_3 to_4 and DCIS_6 to _8 are whole tissue extractions.

Western blot analysis of eukaryotic EV markers (CD9 and CD63) confirmed the absence of cross-contamination between *Lr* EVs and mammalian EVs (Figures 4A and 4B; Data S4). Intact *Lr* EVs stained positive for lipoteichoic acid and S-layer protein, which can be used as surface markers^22^ (Figures 4C and 4D; Data S4). S-layer protein and *Lr* 16S rDNA were detected in the tissue biopsy specimens of DCIS lesions with comedonecrosis or solid features (Figures 4E–4G and S7; Data S4), thus confirming the presence of *Lactobacillus* derived material and possibly EVs in the breast tissue.

These results identified previously unknown *Lactobacillus* EV markers and supported the presence of *Lactobacillus-*derived bioanalytes in breast tissue.

### *Lactobacillus* EVs increase the intracellular calcium levels of BT-474 cell monolayer cultures exposed to short-term calcium stress

**Figure 5:**
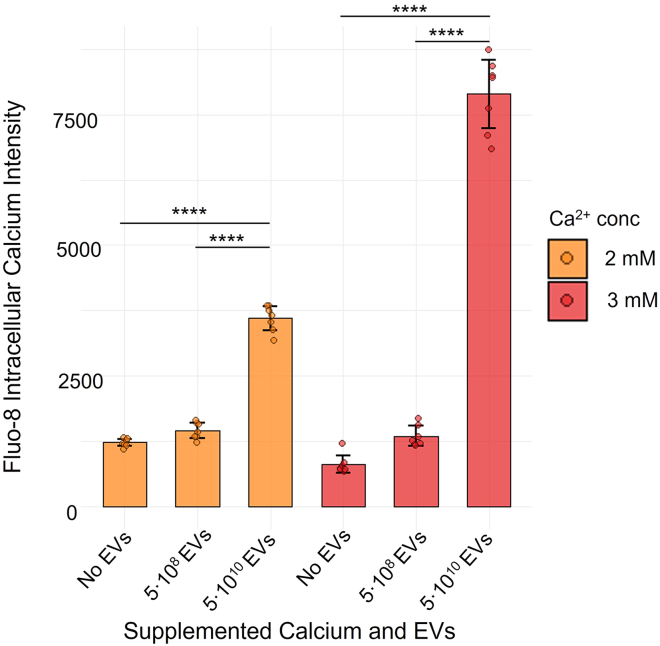
*Lactobacillus* EVs increased intracellular calcium levels in monolayer BT-474 cell cultures exposed to short-term calcium stress *L*. *rhamnosus* EVs (5 × 10^8^ EV and 5·10^10^ EV label, respectively) induced an increase in intracellular calcium in monolayer BT-474 cell cultures faced with a short-term exposure (12 h) to high calcium levels in the medium (2 mM and 3 mM supplemented [Ca2+]). Fluo-8 fluorescence signals were measured at 490/525 nm excitation/emission wavelengths. The no EVs label signifies that no EVs were added to the culture medium. Statistics: Welch two-sample t test; ∗∗∗∗ <0.0001. Data are represented as mean +/− standard deviation.

*Lr* EVs (10^8^ and 10^10^) induced an intracellular calcium increase in BT-474 cell monolayer cultures exposed to short-term (12 h) elevated calcium levels in the medium (2 mM and 3 mM in addition to standard DMEM/F12 medium). Fluo8 staining showed a dose-dependent increase in intracellular calcium levels with both calcium stress and EV levels (Figures 5 and S8). 10^10^ and 10^8^ EVs caused 10- and 1.6--fold increase in intracellular calcium in DMEM/F12 enriched with 3 mM CaCl_2_ (Welch two-sample t test p 2 × 10^-^^9^ and 6 × 10^-5^), and 3- and 1.2--fold increase in non-supplemented DMEM/F12 (Welch two-sample t test p 10^-^^9^ and 0.0001), respectively. These results suggest that *Lr* EVs play a role in elevating intracellular calcium levels in breast cancer cells exposed to short-term elevated extracellular calcium levels.

### 3D BT-474 spheroids: A model of the intraductal hypoxic gradient

**Figure 6:**
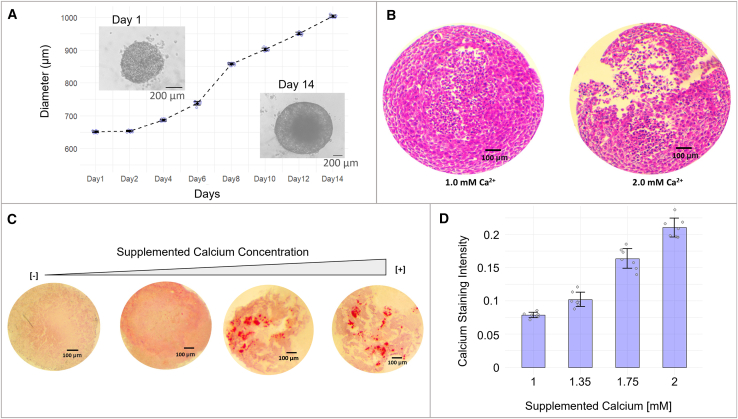
Characterization of BT-474 breast cancer spheroids (A) The spheroid diameter changed over the course of 14 days, with a mean and standard deviation of 9 spheroids. The scale bar is 200 μm. (B) Hematoxylin and eosin (H&E) staining of a spheroid cross-section showing necrotic and hypoxic areas in the central regions when the spheroids reached a diameter of 1000 μm. The scale bar is 100 μm. (C and D) Calcium deposition (Alizarin Red S stain) in BT-474 spheroids increased with increasing amounts of added calcium in the culture media in a dose-dependent manner. Data are represented as mean +/− standard deviation. The mean and standard deviation were calculated for seven spheroids. The scale bar is 100 μm.

To evaluate the effect of *Lr* EVS on calcium modulation and deposition in a breast cancer model under chronic calcium stress, we first developed a 3D spheroid model using BT-474, a HER2-positive breast cancer cell line. After 14 days of 3D culture, the spheroids reached diameters of 800–1000 μm (Figure 6A; Data S4). As expected, necrotic and hypoxic areas (H&E staining) were prominent in the central regions when the spheroids reached a diameter of 1000 μm (Figures 6B; Data S4), which was the limit of the oxygen diffusion distance from the external culture medium.

### Calcium export and deposition within spheroids are dependent on calcium levels in the culture medium

To test the capability of calcium mineralization of the 3D spheroid model, spheroids were exposed to increasing amounts of extracellular [Ca2+] from 1.35 mM up to 2.0 mM. The control group of spheroids was left untreated in a normal growth medium with a standard [Ca2+] of 1.0 mM. The spheroids were supplied with fresh calcium-enhanced medium every 48 h. After 14 days of growth in calcium-enhanced media, the spheroids were fixed and stained with Alizarin Red S dye. Control untreated spheroids demonstrated weak Alizarin Red S staining, indicating little to no formation of calcium deposits (Figure 6C; Data S4). Spheroids treated with 2.0 mM [Ca2+] showed 1.7 times higher calcium export and salt deposition than spheroids treated with 1.0 mM [Ca2+] (Alizarin Red S staining, t test *p* < 0.001, Figure 6D). Taken together, these results show that the spheroid model system is suitable for studying the effect of *Lr* on extracellular calcium precipitate deposition within the tissue microenvironment.

### *L*. *rhamnosus*-derived EVs increase calcium export and calcium salt deposition in a dose-dependent manner within the spheroid exposed to chronic calcium stress

**Figure 7:**
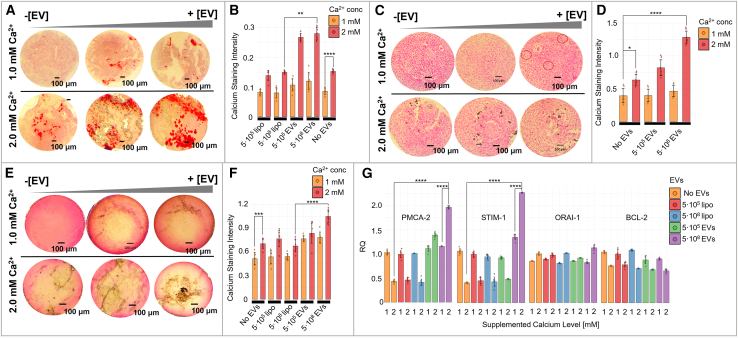
Supplemented calcium and bacterial EVs in the culture media modulate extracellular calcium deposition in BT-474 breast cancer spheroids (A and B) Higher calcium deposits were observed in spheroids exposed to increasing amounts of EVs and calcium in the culture media (whole spheroids Alizarin Red stain), A, Alizarin Red stain, B. The error bars represent the standard deviation; the mean and standard deviation were calculated for seven spheroids. (C) Paraffin-embedded, ethanol-fixed spheroid cross sections show (Von Kossa stain) increased calcium deposition in the central regions when spheroids were exposed to increasing amounts of supplemented calcium and bacterial EVs in the culture medium. (D) Data are represented as mean +/− standard deviation; the mean and standard deviation were calculated for seven spheroids. (E) Whole spheroid show (Von Kossa stain) increased calcium deposition in central regions when spheroids were exposed to increasing amounts of supplemented calcium and bacterial EVs in the culture medium. (F) The error bars represent the standard deviation; the mean and standard deviation were calculated for seven spheroids. (G) The mRNA levels of calcium effluxes/channels PMCA2, ORAI-1, STIM1 and apoptotic marker BCL-2 were measured by real-time quantitative PCR in spheroids exposed to increasing quantities of calcium (1 mM and 2 mM) and bacterial EVs (5×10^5^ EV and 5×10^8^ EV) in the culture media. Liposomes (Ø = 100 nm) were used as controls (5×10^5^ lipo and 5×10^8^ lipo). GAPDH was used as the housekeeping gene and the results were normalized to GAPDH. The error bars represent the standard deviation; the mean and standard deviation were calculated on 7 spheroids. Statistics: Welch two-sample t test; ∗∗∗∗ <0.0001. The scale bar is 100 μm.

To understand how *Lr* EVs affect calcium deposition, spheroids were exposed to increasing concentrations of EVs and extracellular calcium over the course of 2 weeks. Synthetic liposomes were used as internal controls for the EV experiments. Calcium staining intensity of spheroids exposed to 2 mM [Ca2+] as opposed to 1 mM [Ca2+]-containing medium showed an increase in extracellular calcium deposits by 1.8-, 1.5-, and 1.6--fold in Alizarin Red S of whole spheroids, Von Kossa of sectioned spheroids, and Von Kossa of whole spheroids, respectively (t = 9.58 *p* = 8.54 × 10^−6^, t = 2.37 *p* = 0.041, and t = 4.37 *p* = 0.0011, respectively, Figures 7A–7F; Data S4). Spheroids treated with 5×10^8^ *Lr* EVs and 2.0 mM [Ca2+] containing medium showed an increase of 1.94 in Alizarin Red S staining intensity compared to control spheroids exposed to 2 mM [Ca2+]-containing medium (t = 3.52 *p* = 0.0043, Figures 7A and 7B; Data S4). The same behavior was observed using the von Kossa method (Figures 7C–7F; Data S4). When treated with *Lr* EVs, spheroid-associated calcium deposits increased in a dose-dependent manner, with the staining intensity progressively increasing (up to a 2-fold increase) in a manner that was positively related to medium calcium content and EV treatment concentration (Figures 7C–7F, Data S4). Von Kossa staining of sectioned spheroids showed 1.5-fold increase in calcium staining intensity induced by 5×10^8^ *Lr* EVs and 2.0 mM [Ca2+] treatment with respect to 2.0 mM [Ca2+] liposome control (t = 12.66 *p* = 3.16 × 10^−8^, Figures 7C and 7D; Data S4). The fold change for non-sectioned whole spheroids was 2 (t = 6.99 *p* = 2.02 × 10^−5^, Figures 7E and 7F; Data S4). Taken together, these results suggest that *Lr* -derived EVs play a role in increasing extracellular calcium deposition in breast cancer cells exposed to high levels of extracellular calcium stress.

### *L*. *rhamnosus*-derived-EV treatment is associated with overexpression of PMCA2 and STIM1

To understand the molecular mechanism underlying the influence of *Lr* EVs on calcium handling in breast cancer cells, we performed a real-time RT-PCR study of several genes, including PMCA2, calcium channel ORAI-1, calcium sensor STIM1, and apoptotic marker BCL-2, to determine whether their expression varied upon exposure to bacterial EVs (Figure 7G). Although not statistically significant, ORAI-1 mRNA expression tended to be higher in spheroids exposed to elevated calcium and EV concentrations. BCL-2 mRNA levels decreased slightly with increasing [Ca2+] concentrations and EV quantities. PMCA2 and STIM1 mRNA levels were reduced by approximately 50% in spheroids exposed to 2.0 mM [Ca2+] compared to those exposed to 1.0 mM [Ca2+]. In the presence of 2 mM calcium, PMCA2 and STIM1 mRNA expression in spheroids treated with 10^8^ EV was markedly elevated by 4.75-fold (t test *p*-value = 3.2 · 10^−11^) and 5.50-fold (t test *p* value = 8.3 · 10^−7^), respectively, as compared to EV-untreated spheroids. The gene expression modulation was EV- and calcium supplementation-dose dependent.

## Discussion

Experimental evidence supporting the potential functional role of the microbiome and its EV products in breast intraductal space is limited. The concept that the intraductal microbiome may play a role in calcium modulation is supported by the importance of calcium handling during lactation^14^^,^^23^ when milk is known to contain a resident microbiome. Such resident intraductal microbiome populations may play a role in physiological calcium regulation during lactation, which may be carried over following the transformation of the ductal epithelium into pre-invasive and invasive carcinomas. In this study, we used anonymous human breast cancer biospecimens, and 2D and 3D culture models to collect functional data to support the influence of bacterial EV on intracellular calcium homeostasis and calcium export/deposition in breast cancer cells.

*L. rhamnosus* (*Lr*) is a milk-resident bacterium that modulates calcium handling. *Lr* can regulate various calcium salt formations during milk production, including calcium phosphate, calcium chloride, calcium citrate, and calcium carbonate,^24^ which are diverse sources for calcium hydroxyapatite, the main component of intraductal microcalcifications.^25^ Microcalcifications are an important feature of mammography, and are frequently associated with malignant breast cancer.^26^ In our proteomic study, *Lr* was more abundant in the serum and plasma of women with breast cancer than in benign controls. From the perspective of breast cancer, a higher abundance of *Lr* is of particular interest because of its known influence on calcium regulation. It has been shown that HER2 positive cancers have a higher incidence of microcalcifications.^15^ As shown in Figure S4, the *Lactobacillus* serum proteomic signature was higher in HER2 positive cases and *Lr* peptide hits increased in patients with higher BI-RADS scores. We have previously shown that the HER2 receptor is intimately associated with PMCA2, the major calcium export channel in the cell membrane.^15^ Therefore, among the broad repertoire of microbiota species detected in blood products and saliva, *Lr* was chosen as a model organism to study the influence of the microbiome on calcium handling in breast cancer. Consistent with this connection between *Lr*, HER2, and calcium handling, our *in vitro* spheroid data (Figure 7G) showed that chronic exposure to calcium stress upregulated the transcript levels of PMCA2 in HER2 positive breast cancer spheroids, and that this adaptation mechanism is modulated in a dose-dependent manner by treatment with *Lr* EVs.

This study introduces a novel role for *Lr* EVs as functional mediators. We provide a complete characterization of the proteome of *Lr* EVs, addressing the otherwise limited knowledge of bacteria-derived EV molecular payloads.^27^ The surface markers (lipoteichoic acid and S-layer protein) and proteins identified in lysed *Lr* EVs in the present study can serve as EV markers of *L*. *rhamnosus* or gram-positive bacteria. Proteomic analysis of *Lr* EVs revealed new molecular information and provided insights into the mechanisms by which *Lr* influences calcium signaling in cancer cells. Enzymes involved in glycolysis and pentose phosphate and hexosamine biosynthesis pathways are of particular interest because energy metabolism pathways have been shown to influence [Ca2+] dynamics.^9^

The presence of nucleic acids in *Lr* EVs raises the question of their bioactivity and possible role in modifying recipient eukaryotic cell translation. While a large body of research supports the presence of functional miRNAs in eukaryotic EVs and EV-associated miRNA capability to post-transcriptionally regulate target mRNA in eukaryotic recipient cells,^28^^,^^29^^,^^30^ the question of whether this form of regulation is possible between microbiota and eukaryotic cells remains largely unanswered. miRNA-sized sRNAs have been detected in outer membrane vesicles of periodontal pathogens and have been shown to enter host cells and modulate specific host protein levels.^20^^,^^31^ Further research is warranted to fully elucidate the molecular cargo of *Lr* EVs and to investigate their effects on the expression of STIM1, PMCA2, and other genes involved in intracellular calcium homeostasis.

Emerging evidence suggests that microbiome EVs can penetrate tumor tissues, as observed in pancreatic cancer.^21^ In this study, the ability of *Lr* EVs to infiltrate tumor tissues was confirmed using patient biopsy samples and EV-specific markers. S-layer protein (Slp),^32^ a marker of intact *Lr* EVs, and *Lactobacillus* DNA were identified in laser-microdissected high-grade human intraductal carcinoma *in situ* (DCIS) lesions.

Intracellular calcium homeostasis and extracellular calcium export regulate breast cancer cell survival under calcium stress and premalignant progression, differentiation, migration, and invasion. Persistent elevation of resting intracellular [Ca2+] levels augments malignant potential by influencing gene expression, facilitating cell migration, and promoting proliferation, thereby initiating and sustaining tumor growth.^33^^,^^34^ We previously documented a direct phenotypic role of calcium export through PMCA2 channels linked to HER2 function and BT474 breast cancer cell survival under calcium stress.^16^ Intraductal macro and microcalcifications are common radiologic and histopathologic features of high-grade pre-invasive DCIS,^25^ particularly in lesions associated with central hypoxic necrosis. However, the molecular role of extracellular calcifications in stimulating or suppressing breast cancer remains poorly understood.^35^ One hypothesis is that calcium export and extracellular precipitation are homeostatic mechanisms used by cancer cells to survive in the hypoxic nutrient-deprived intraductal space under calcium stress. Following invasion into the periductal space and subsequent tumor vascularization and growth, the functional role of microbiome EVs in influencing cancer progression may change because the microenvironment is markedly different. The findings herein provide tools to study the role of microbiome EVs in future studies on tumor progression beyond the premalignant stage. *Lr* EVs induced increased calcium uptake in monolayer cell cultures exposed to short term calcium stress. Overnight exposure to *Lactobacillus* EVs (with no viable organisms) caused an increase in the intracellular calcium levels of BT-474 cells in a calcium concentration–dependent manner, compared to liposome controls. These findings would support the role of *Lactobacillus* EVs in contributing to the high intracellular calcium levels in breast cancer cells. In turn, this promotes cell proliferation, migration, tumor growth, and malignant phenotypes.^33^^,^^34^

A 3D breast cancer spheroid model of an intraductal hypoxic gradient was used to study the biomineralization pathways. Eukaryotic cells typically live within a narrow range of intracellular calcium levels. Cells that survive under constantly elevated exogenous calcium stress can do so by elevating calcium export channels.^15^^,^^16^ It has been documented that the mammary ductal epithelium is uniquely adapted to manage the high concentrations of calcium necessary for exporting high concentrations of calcium into milk during lactation.^16^ We hypothesized that these physiological functions are retained by the transformed breast epithelium. It has been previously established that cancer spheroid cultures can mimic the main features of solid tumors, including structure, cell layer organization, hypoxia, and nutrient gradients.^36^ Vidavsky et al. utilized 3D spheroid models to study biomineralization pathways in breast cancer microcalcifications and found that the upregulation of mineralization in cancer cells is associated with malignancy. Moreover, calcium deposition within the spheroid can be readily induced at the center of the spheroid to a level that is higher for elevated doses of extracellular calcium in the culture medium. Thus, spheroid culture simulates several important features of carcinogenesis within the intraductal space.^37^ As neoplastic breast epithelial cells accumulate and proliferate within the intraductal space, the duct diameter expands and the cells within the center of the duct are placed at a growing distance from the surface of the duct. These central cells become hypoxic and undergo metabolic calcium stress (from the dying cells around them leaking calcium) because the blood vessels supplying oxygen and nutrients are outside the duct perimeter. The geometric oxygen and nutrient gradient observed in spheroid culture upon cross-section is a good functional model of human intraductal carcinoma, as noted by Vidavsky.^36^ The relative diameter of the spheroid and carcinoma cell packing density are similar to those of human DCIS lesions because both the spheroid and intraductal space are subject to the same physical diffusion limitations of oxygen diffusion from the outside.^25^ The tumor cell densities observed in the cross-sections of the spheroids as well as the form, location, and chemical staining characteristics of the calcium extracellular precipitates in the present study were remarkably similar to the cytomorphology and calcium precipitates designated as intraductal microcalcifications in DCIS *in vivo*. *In vivo* microcalcifications are smaller than 0.5 mm and are often associated with hypoxic central necrosis of comedo-type high-grade DCIS.

*Lr* EVs enhanced the survival of breast cancer cells exposed to long-term calcium stress via PMCA2 mediated calcium export. To understand the molecular mechanism underlying this phenomenon, we measured the mRNA expression of PMCA2 and other calcium channels STIM1, ORAI-1 because of their roles in calcium homeostasis and breast cancer survival and progression. PMCA2 appears early in DCIS and is closely associated with HER2.^15^^,^^16^ HER2 overexpression is strongly associated with the induction of microcalcifications in HER2-positive patients.^13^ In previous studies, PMCA2 activity was found to maintain low intracellular calcium levels, which are necessary for HER2 signaling and resistance to endocytosis.^15^ Conversely, PMCA2 knockout increased intracellular calcium levels, inhibited HER2 signaling, and led to HER2 internalization and degradation in breast cancer cells. Silencing PMCA2 reduces breast cancer cell proliferation and sensitizes cancer cells to the cytotoxic agent doxorubicin.^16^ The STIM1-ORAI-1 complex also appears early in DCIS and plays an important role in the development and progression.^11^^,^^15^^,^^16^ In a previous study, the overexpression of STIM1 and ORAI-1 increased the store-operated calcium entry process and led to cell migration, metastasis, and invasion.^11^ Single nucleotide variants in STIM1 are associated with an increased risk of tumor progression in patients with HER2-positive breast cancer.^38^ Knockdown of STIM1 via RNA interference decreases tumor metastasis in breast cancer.^39^ Our results demonstrated that PMCA2, ORAI-1, and STIM1 were differentially expressed in the presence of *Lr* EV stimulation, suggesting a potential role for bacterial EVs in influencing the differentiation, migration, and metastasis of breast cancer cells.

In Jeong et al.,^15^ we previously used external concentrations of calcium ranging from 2 to 20 mM to induce calcium stress in cultured monolayers of BT474 HER2+ breast cancer cells. Jeong et al. indicated that these cells could survive at high calcium concentrations in the medium by pumping calcium out via PMCA2 channels. In the present study, we used the same human breast cancer cell type, grown in a monolayer or spheroid. Knockdown of PMCA2 by Jeong et al. resulted in a 5-fold increase in intracellular calcium levels under calcium stress. Consistent with these previous data, BT474 cells survived high external calcium stress by exporting calcium via PMCA2 in the present study. Herein, we also showed that translating the same model from a 2D to a 3D state resulted in calcium precipitate accumulation in the extracellular space of spheroids. Furthermore, by extending this concept to the present model, spheroid cells adapted to chronic increased calcium stress by upregulating (increased translation) PMCA2, which was augmented in the presence of *Lr* EVs. Thus, EVs augment the ability of cells to adapt and survive under increased calcium stress by pumping out calcium and depositing it in the extracellular space. The alterations in ORAI-1 and STIM1 transcription were also consistent with the desensitization and adaptation of spheroid cells to survive under high external chronic calcium challenge. Under acute monolayer exposure of the cells to calcium stress in the presence of *Lr* EVs, it was noted that the EVs induced a significantly elevated burst of cytoplasmic calcium in an EV dose-dependent manner. When we translate this to the continuous chronic exposure of the growing spheroids to EVs and calcium stress, a logical interpretation is that EVs increase calcium influx, leading to a necessary higher level of adaptation and homeostasis of the spheroid cells employing an altered baseline of transcription of PMCA2, ORAI-1, and STIM1.

**Figure 8:**
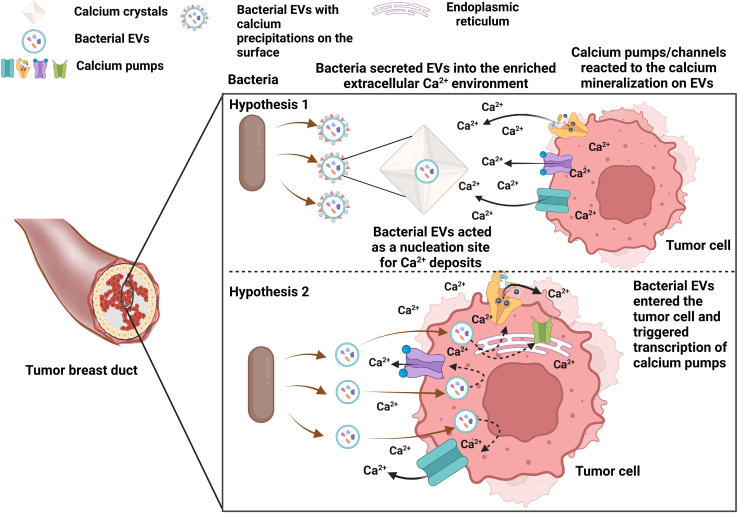
Cartoons depicting the microbiome EV influence hypotheses The first hypothesis is that bacterial EVs act as nucleation sites for extracellular mineral calcium deposition. The second hypothesis is that bacterial EVs are taken up by breast cancer cells and trigger the expression of calcium pumps and channels, thus positively contributing to intracellular calcium homeostasis and hence to the survival, differentiation, migration, and metastatic process of breast cancer cells. Created in https://BioRender.com.

We propose two hypotheses to explain the effects of bacterial EVs on calcium handling in breast cancer (Figure 8). The first hypothesis is that bacterial EVs act as nucleation sites for mineral [Ca2+] precipitation in the extracellular environment. According to this hypothesis, the presence of calcium salt crystals in the tumor environment stimulates calcium pump/channel gene transcription in neighboring breast tumor cells to release more [Ca2+] into the extracellular environment. In support of this hypothesis, literature shows that EVs (outer membrane vesicles) derived from *Escherichia coli* contain calcium-phosphate precipitates on their surface that initiate mineral salt deposition.^40^ The second hypothesis is that bacterial EVs are internalized by breast cancer cells and their molecular content triggers calcium pump/channel gene transcription and calcium export. It has been previously shown that microbiome EVs can be taken up by gut lumen epithelial cells.^9^ The data presented herein supports the second hypothesis. Bacterial EVs continuously present in growing premalignant lesions may chronically induce cancer cells to achieve a higher level of adapted calcium management. In particular, one important adaptation mechanism is the induced transcription of PMCA2 calcium export channels, enabling tumor cells to reduce intracellular calcium to non-toxic levels.^11^^,^^15^^,^^16^ In conclusion, the experimental workflow established in this study provides a new foundation for the study and therapeutic modulation of the functional influence of microbiome EVs on calcium regulation in breast cancer carcinogenesis and the survival of emerging genetically unstable invasive cancer cells within the intraductal space, an important driver of pathogenesis.

### Limitations of the study

This study has several limitations. Only one microbiome species was explored. *Lr* was prioritized on the basis of its preferential association with cancer patients in our clinical study and previous evidence supporting its capability to regulate calcium salts during lactation.^24^ Despite this weakness, our spheroid model provides a reproducible quantifiable system for measuring the functional influence of bacterial EVs on spheroid biology and calcium export channel regulation. This model can serve as the basis for studying the EVs of other microbiome species and exploring treatments that modulate their effects. Empty synthetic liposomes were selected as controls to determine whether the observed effects were due to the *Lr* EV molecular payload rather than the lipid membrane. The comparison of *Lr* EV induced effects with those of other bacterial EVs will help to reveal key molecular effectors, which will be the focus of future research. Although only two *Lr* EV concentrations (10^8^ and 10^10^ EV/mL) were tested, the observed effect at both levels suggested a robust effect of EV-induced calcium modulation. These concentrations were selected based on previous studies to ensure their biological relevance.^41^^,^^42^^,^^43^^,^^44^^,^^45^ We acknowledge that expanding the range of EV concentrations could further refine the dose-response relationship. Future studies should explore a broader spectrum of doses to delineate the precise dynamics and minimum quantity of EV necessary to elicit a response. We used histopathological evidence of hypoxic necrosis in spheroid characterization; however, we did not measure the oxygen gradient itself because this has been recorded in previous studies.^46^^,^^47^^,^^48^^,^^49^ The histopathological features of DCIS and the characteristics of calcium deposits that stain positively with calcium stains commonly used in human pathology are remarkably similar to those observed in 3D spheroids, thus justifying this intraductal calcium stress model. The individual EV molecular components responsible for calcium perturbations induced by EVs were not disambiguated. The comparison of *Lr* EV induced effects with those of other bacteria EVs will help reveal key molecular effectors, which will be the focus of future research. The functional molecular cargo of bacterial EVs can be further characterized by extending the investigation to include carbohydrates, glycolipids, and nucleic acids. The functional roles of individual EV proteins can be ascertained by bacterial protein knockdown or by recombinant protein synthesis. To further explore the alteration in calcium export triggered by bacterial EVs, full transcriptomics before and after exposure to bacterial EVs can be combined with knockdown of calcium regulation genes. Fluorescent labeling of EVs can be used to aid in monitoring cellular uptake and clarify whether internalization is necessary to trigger calcium export and deposition. Although supported by causal evidence in different cell models,^14^^,^^50^^,^^51^ the effects of manipulating STIM1 and PMCA2 gene expression levels on the microcalcification deposition process in BT474 cells were not explored in this study. By regulating intracellular calcium concentration, STIM1 and PMCA2 contribute to calcium extrusion. A higher extracellular calcium ion concentration leads to microcalcifications in the osteogenic environment.^14^ Gene expression silencing and overexpression studies are warranted to confirm the roles of STIM1 and PMCA2 in the Her2+ BT474 cell model. The modulation of STIM1 and PMCA2 by *Lr* EVs was demonstrated at the gene expression level. Further validation is required at the protein level, including the assessment of their activity, cellular localization, and post-translational modifications (PTMs). A more comprehensive understanding of the impact of bacterial EVs on calcium signaling networks will be obtained by studying other regulators, including the sodium-calcium exchanger, NCX, calmodulin, inositol 1,4,5-trisphosphate receptor, IP3R, and ryanodine receptor, RYR.

## Resource availability

### Lead contact

Further information and requests for resources and reagents should be directed to and will be fulfilled by Alessandra Luchini (aluchini@gmu.edu).

### Materials availability

The study did not generate new unique reagents.

### Data and code availability

• Original immunoblotting and microscopy data reported in the paper are contained in Data S4. Mass spectrometry data associated with the manuscript has been deposited in the ProteomeXchange consortium member MassIVE (Accession Code: PXD061715).
• No original code was developed by this study.
• Any additional information required to reanalyze the data reported in this paper is available from the lead contact upon request.

## Acknowledgments

This study was supported by the Center for Applied Proteomics and Molecular Medicine from 10.13039/100006369George Mason University, funded by the Sentara Dorothy G. Hoefer Comprehensive Breast Cancer Center and by the 10.13039/100000002NIH grant number R21AI154295. We thank the staff at the Sentara Dorothy G. Hoefer Comprehensive Breast Center for their support with sample collection and data review. Mr. Jain, Drs. Pierobon and Bishop have provided valuable comments and suggestions.

## Author contributions

R.H., A.L., V.E., and L.A.L. designed this study. R.H. provided human biospecimens, clinical and demographic information, and expert advice to interpret results. N.V., M.A., and I.A. performed 3D spheroid model and cell experiments. F.A., M.H., and M.V.H. prepared bacterial EVs. A.H.S. and Ahana Byne performed the TEM experiments, B.B. designed the PCR primers, and B.B. and N.V. performed real-time PCR analysis. P.G., T.R.P., R.I.A., and C.J.S. performed tissue analysis. N.V. and W.Z. performed sample preparation and mass spectrometry analysis. N.V., A.L., V.E., and R.P.A. analyzed the data. N.V. wrote the manuscript. A.L., V.E. and L. L. reviewed and edited the manuscript. All the authors have read and approved the final manuscript.

## Declaration of interests

The authors declared the following potential conflicts of interest with respect to the research, authorship, and/or publication of this article: V.E., L.L., and A.L. are coinventors in granted patents US 9,012,240 and US 8,497,137, related to the affinity particles. The patents are owned by the George Mason Research Foundation. Ceres Nanosciences licensed the rights of the patents. L.L. and A.L. own shares of Ceres Nanosciences. The remaining authors declared no competing interests.

## STAR★Methods

### Key resources table

**Table:** 

REAGENT or RESOURCE	SOURCE	IDENTIFIER
**Antibodies**		

Anti-CD63 antibody [MEM-259]	Abcam	Cat# ab8219; RRID: AB_306364
Anti-CD9 antibody [MEM-61]	Abcam	Cat# ab2215; RRID: AB_302894
Anti-beta Actin antibody [mAbcam 8226] - Loading Control	Abcam	Cat# ab8226; RRID: AB_306371
Goat Anti-Mouse IgG H&L (HRP)	Abcam	Cat# ab97023; RRID: AB_10679675
Anti-Staphylococcus aureus LTA antibody, Mouse monoclonal	Sigma-Aldrich	SAB4200883
Surface Layer Protein (AA 301–400) antibody (HRP)	antibodies-online	ABIN2179511

**Bacterial and virus strains**		

*Lactobacillus rhamnosus* LMS-2 strain	BEI Resources, VA, USA	LMS-2 strain

**Biological samples**		

Plasma	Sentara Dorothy G. Hoefer Comprehensive Breast Center (Newport News, VA, USA)	Sentara and George Mason University IRBs (GMU IrbNet 978106-1).
serum	Sentara Dorothy G. Hoefer Comprehensive Breast Center (Newport News, VA, USA)	Sentara and George Mason University IRBs (GMU IrbNet 978106-1).
saliva	Sentara Dorothy G. Hoefer Comprehensive Breast Center (Newport News, VA, USA)	Sentara and George Mason University IRBs (GMU IrbNet 978106-1).
Tissue biopsy	Sentara Dorothy G. Hoefer Comprehensive Breast Center (Newport News, VA, USA)	Sentara and George Mason University IRBs (GMU IrbNet 978106-1).

**Chemicals, peptides, and recombinant proteins**		

N-isopropylacrylamide (NIPAM)	Sigma-Aldrich	415324-50G
N,N′-methylenebisacrylamide (BIS)	Sigma-Aldrich	110-26-9
acrylic acid (AAc)	Sigma-Aldrich	38862-24-7
allylamine (AA)	Sigma-Aldrich	145831
Reactive Blue 221	OrganicDyes and Pigments	93051-41-3
Trypan Blue	Sigma-Aldrich	72-57-1
Bismarck Brown Y	Sigma-Aldrich	10114-58-6
Vinyl sulfonic acid (VSA)	Sigma-Aldrich	9002-97-5
Detergent Removal Spin Column	Pierce™; Thermo Fisher Scientific	87780
C18 Spin Column	Pierce™; Thermo Fisher Scientific	87777
MRS broth	Sigma-Aldrich	41782-500G-F
Liposomes (L-alpha phosphatidylcholine and cholesterol control, Ø = 100 nm)	Creative Biolabs, Inc., NY, USA	LDLY-0123-LY124
Dulbecco’s modified Eagle medium: Nutrient Mixture F-12 medium (DMEM/F12)	Thermo Fisher Scientific, MA, USA	21331020
fetal bovine serum qualified USA (Gibco)	Thermo Fisher Scientific, MA, USA	26140079
penicillin-streptomycin (Gibco)	Thermo Fisher Scientific, MA, USA	15140122
L-glutamine	Thermo Fisher Scientific, MA, USA	A2916801
Trizol	Thermo Fisher Scientific, MA, USA	15596026
iScript™ cDNA Synthesis Kit	Bio-Rad	1708890
SsoAdvanced Universal SYBR Green Supermix	Bio-Rad	1725271
Nuclear Fast Red	Sigma-Aldrich	N3020
Alizarin Red	Thermo Fisher Scientific, MA, USA	400481000

**Critical commercial assays**		

Fluo-8 Calcium Flux Assay Kit	Abcam	ab112129

**Deposited data**		

Proteomics data	MASSive/ProteomeXchange	PXD061715 Calcium Handling in Breast Cancer

**Experimental models: Cell lines**		

Human breast cancer cell line BT-474	ATCC, USA	HTB-20

**Oligonucleotides**		

bacterial 16S rDNA-Forward	IDT DNA	TCCTACGGGAGGCAGCAGT
bacterial 16S rDNA-Reverse	IDT DNA	GGACTACCAGGGTATCTAATCCTGTT
L. rhamnosus-specific 16S rDNA Forward	IDT DNA	GGCGTGCCTAATACATGCAA
L. rhamnosus-specific 16S rDNA Reverse	IDT DNA	GTCCGCCACTCGTTCAAAA
PMCA2 Forward	IDT DNA	TCCTCAACGAACTCACCTGC
PMCA2 Reverse	IDT DNA	GCCGTGTTGATATTGTCGCC
BCL-2 Forward	IDT DNA	GGATAACGGAGGCTGGGATG
BCL2 Reverse	IDT DNA	GGCCAAACTGAGCAGAGTCT
ORAI-1 Forward	IDT DNA	GCTCTGCTGGGTCAAGTTCT
ORAI-1 Reverse	IDT DNA	CGTTGAGCTCCTGGAACTGT
STIM1 Forward	IDT DNA	CCATCACCACTACCACCACC
STIM1 Reverse	IDT DNA	CCAAGCTCTCTGAATGCCCA
GAPDH Forward	IDT DNA	GTTCGACAGTCAGCCGCATC
GAPDH Reverse	IDT DNA	GGAATTTGCCATGGGTGGA

**Software and algorithms**		

Interactive Tree of Life (iTOL)		
Perseus software	Max Planck Institute of Biochemistry, Germany	
ImageJ	NIH, USA	
BioRender	Ontario, Canada	
PEAKS-X Studio	Bioinformatics Solutions Inc., Canada	
NCBI resourses	NIH, USA	

### Experimental model and study participant details

#### Humans

Plasma, serum, saliva, and tissue biopsy samples were collected from 150 female participants undergoing diagnostic biopsies after suspicious mammogram results at Sentara Dorothy G. Hoefer Comprehensive Breast Center (Newport News, VA, USA). Informed consent was obtained from all participants. The study was approved by the Institutional Review Boards of Sentara Healthcare Systems and George Mason University (GMU IRB, IrbNet #978106-1). Clinical data including age, family history of breast cancer, lesion size, tumor type, and Breast Imaging Reporting and Data System (BI-RADS) radiographic risk scores were collected and summarized in Tables 1 and 2. All study participants were female. Information regarding ancestry, race, ethnicity, and socioeconomic status was not available due to IRB restriction. The influence of these variables on the results of the study was not assessed, which may limit the generalizability of the findings. All experiments involving human samples were approved by the Institutional Review Boards of Sentara Healthcare Systems and George Mason University (GMU IRB approval: IrbNet 978106-1).

#### Cell lines

The human breast cancer cell line BT-474 was obtained from ATCC, USA (catalog number HTB-20). ATCC reports the following information regarding the sex of cells: the BT-474 line was isolated from a solid, invasive ductal carcinoma of the breast of a 60 years old white female patient. Cells were cultured at 37°C, 5% CO_2_, in Dulbecco’s Modified Eagle Medium/Nutrient Mixture F-12 (DMEM/F12) supplemented with 10% heat-inactivated exosome-free fetal bovine serum (FBS), 1% penicillin-streptomycin, and 1% L-glutamine (2 mM; all from Thermo Fisher Scientific, MA, USA). Cells were used within 6 months form purchase from ATCC and cell authentication was not performed.

### Method details

#### Affinity hydrogel particle synthesis

Hydrogel nanoparticles composed of poly(N-isopropylacrylamide-co-acrylic acid) [poly(NIPAm-co-AAc)] and poly(N-isopropylacrylamide-co-allylamine) [poly(NIPAm-co-AA)] were synthesized by precipitation polymerization.^52^ Poly(NIPAm-co-AAc) nanoparticles were synthesized by dissolving N-isopropylacrylamide (NIPAm; Sigma-Aldrich, USA), N,N′-methylenebisacrylamide (BIS; Sigma-Aldrich, USA), and acrylic acid (AAc; Sigma-Aldrich, USA) in water, followed by nitrogen purging and polymerization initiated with potassium persulfate (KPS; Sigma-Aldrich, USA) at 70°C for 6 h. Resulting nanoparticles were functionalized by amidation chemistry with amine-containing dyes such as Reactive Blue 221 (Organic Dyes and Pigments), Trypan Blue, or Bismarck Brown Y (both from Sigma-Aldrich, USA) after activation with N-(3-dimethylaminopropyl)-N′-ethyl carbodiimide hydrochloride (EDC; Fluka Analytical) and N-hydroxysuccinimide (NHS; Sigma-Aldrich, USA).^52^ A vinyl sulfonic acid (VSA; Sigma-Aldrich, USA)-containing shell was added to Bismarck Brown Y-functionalized particles via a secondary polymerization reaction to enhance their size-selective sieving capabilities.^52^ Affinity hydrogel particle batches were stored at 4°C and checked for bacterial contamination by agar cultures every six months and before use.

#### Affinity particle sample enrichment

Biospecimens were obtained following procedures approved by the Institutional Review Boards of Sentara Healthcare Systems and George Mason University (GMU IRB, IrbNet #978106-1). Plasma, serum, and saliva samples were de-identified at Sentara, frozen at −80°C, and sent to George Mason University for analysis. Plasma and serum samples (500 μL) were diluted 1:2 with 50 mM Tris-HCl, pH 7.0, while saliva samples were used without dilution. Samples were spiked with 300 ng lysozyme (Sigma-Aldrich, USA) as an internal control. Affinity particles (200 μL Bismarck Brown Y-VSA, 150 μL Reactive Blue 221, and 150 μL Trypan Blue; each at 5 mg/mL dry weight suspension) were incubated with samples for 60 min. Particles were separated by centrifugation (16,100 × g, 15 min, room temperature), washed thrice with MilliQ water, and eluted in 20 μL elution buffer (4% sodium dodecyl sulfate (SDS), 50mM NH_4_HCO_3_, pH 7.8). Eluates were treated with Detergent Removal Spin Column (Pierce; Thermo Fisher Scientific, USA) according to the manufacturer’s instructions. Proteins were reduced with 200 mM dithiothreitol, alkylated with 50 mM iodoacetamide, and digested with trypsin (1:20 protease: protein w/w ratio) overnight at 37°C. The peptides were purified using a C18 Spin Column (Pierce™; Thermo Fisher Scientific, USA) and then dried under nitrogen flow.

#### Mass spectrometry analysis

LC-MS/MS experiments were performed using an Orbitrap Fusion system (Thermo Fisher Scientific, MA, USA) equipped with a nanospray EASY-nLC 1200 HPLC system (Thermo Fisher Scientific). The peptides were separated using a reversed-phase PepMap RSLC 75 μm i.d. × 15 cm column with a 2 μm C18 resin LC column (Thermo Fisher Scientific, MA, USA). The mobile phase consisted of 0.1% aqueous formic acid (mobile phase A) and 0.1% formic acid in 80% acetonitrile (mobile phase B). After sample injection, the peptides were eluted by using a linear gradient from 5% to 50% B over 90 min and ramped to 100% B for an additional 2 min. The flow rate was set at 300 L/min. The Orbitrap Fusion was operated in data-dependent mode in which one full MS scan (60,000 resolving power) from 300 Da to 1500 Da using quadrupole isolation was followed by MS/MS scans in which the most abundant molecular ions were dynamically selected by Top Speed and fragmented by collision-induced dissociation using a normalized collision energy of 35%.

#### Mass spectrometry bioinformatics analysis

Tandem mass spectra were searched against a microbiome database retrieved from the National Center for Biotechnology Information (NCBI) using PEAKS-X Studio (Bioinformatics Solutions Inc., Canada). The microbiome database was obtained by retrieving the NCBI protein database for organisms listed in Table S5, including *Veillonella dispar*, *Bacteroides* sp., *Fusobacterium* sp., *Parabacteroides distasonis*, *Megamonas* sp., *Lactobacillus* sp., and other bacteria that belong to the human microbiome, and have been identified in breast tissue samples in previous studies.^2^^,^^3^^,^^6^^,^^17^ Tryptic cleavage constraints were used: mass tolerance for precursor ions was 5 ppm, and mass tolerance for fragment ions was 0.5 Da. Data were analyzed with oxidation (+15.9949 Da) of methionine as a variable post-translation modification and carbamidomethyl cysteine (+57.0215) as a fixed modification. A 1% false discovery rate was used as the cut-off value for reporting peptide spectrum matches (PSM) from the database. A series of BLAST analyses were performed to authenticate peptides that are unique to an organism at the genus or species level.^53^ Peptides were authenticated if all the following requirements were met: 1) sequence was longer than 7 amino acids to minimize the probability of random attribution, 2) 100% homology, 100% coverage, Evalue <10^4^ with one organism at the genus or species level, and 3) less than 90% homology with all other organisms in the non-redundant database. One organism was identified if two unique peptides were detected.^53^^,^^54^^,^^55^ The *Lr* EV proteome was obtained by searching the tandem mass spectra against the NCBI *Lactobacillus rhamnosus* database using Peaks-X Studio.

#### EV isolation and characterization

The *Lactobacillus rhamnosus* LMS-2 strain (BEI Resources, VA, USA) was cultured at 37°C in 100 mL of de Man, Rogosa, and Sharpe broth (10^8^ CFU/mL, MRS, BD Difco, MI, USA). MRS broth was autoclaved and filtered through 0.22-μm filters before use. To separate EVs from the bacterial culture, 100 mL of the bacterial suspension was centrifuged at 5000 × g for 15 min at 4°C to pellet the bacterial cells. The supernatants were then collected, filtered two times with 0.22-μm filter and subjected to ultracentrifugation at 100,000 × g for 90 min at 4°C (Ultracentrifuge WX Ultra 80, Thermo Fisher Scientific, MA, USA). The supernatant was discarded, and the pellet was washed in PBS 1X, and ultracentrifuged again at 100,000 × g for 90 min at 4°C. EV size and concentration were measured using ZetaView® (Analytik Ltd, UK), and vesicles were characterized via Transmission Electron Microscopy (TEM).^56^

#### Immunoblot analysis

Total protein from EVs was extracted using RIPA lysis buffer (Thermo Fisher Scientific, MA, USA). The protein concentration of the EVs was determined using the Bradford assay. To perform western blot analysis, 10 μL of EVs sample was heated for 5 min before being separated on a Novex™ WedgeWell™ 4–20%, Tris-Glycine gel (Thermo Fisher Scientific, MA, USA) at 120 V for 90 min. The gel was transferred to 0.45 μm nitrocellulose membranes (Bio-Rad Laboratories, CA, USA), and membranes were blocked in 5% skim milk diluted in Tris-buffered saline containing 1% Tween 20 for 1 h at room temperature. The membrane was then incubated with the appropriate primary antibodies diluted in Tris-buffered saline containing 5% skim milk and 1% Tween 20 (anti-CD63 mouse monoclonal antibody, 1:1000; Abcam, UK), anti-CD9 mouse monoclonal antibody (1:1000; Abcam, UK), and anti-beta actin mouse monoclonal antibody (1:1000; Abcam, UK). Membranes were then incubated with a horseradish peroxidase (HRP)-conjugated secondary antibody (1:10,000, Abcam, UK) for 1 h at room temperature and developed using an enhanced chemiluminescence kit (ECL, Thermo Scientific). Luminescence was visualized using an Azure c300 gel imaging system (Azure Biosystems). For the dot blot analysis, bacterial material and lysed human biopsy material were spotted onto a nitrocellulose membrane that was blocked as described above. The Anti-lipoteichoic acid (Sigma #SAB4200883) and anti-S-layer protein (Antibodies-online.com #ABIN2179511) antibodies were diluted 1:400 and 1:300 in Tris-buffered saline supplemented with 5% skim milk and 1% Tween 20, respectively. The signals were detected using an enhanced chemiluminescence (ECL) kit.

#### 2D cell culture conditions

The human breast cancer cell line BT-474 was purchased from American Type Culture Collection (ATCC, VA, USA) and grown in Dulbecco’s modified Eagle medium: Nutrient Mixture F-12 medium (DMEM/F12, Thermo Fisher Scientific, MA, USA) supplemented with 10% heat-inactivated exosome-free fetal bovine serum, 1% penicillin-streptomycin, and 1% L-glutamine 2mM (Thermo Fisher Scientific, MA, USA). Cells were cultured in standard T75 flasks at 37°C, 5% CO_2_, and 90% humidity. The medium was replaced every 72 h. Cells were maintained in 2D culture for no more than 10 consecutive passages before being discarded.

#### Fluo8 intracellular calcium staining

BT-474 cells were seeded at a density of 35,000 cells/100 μL of serum-free DMEM/F-12 medium per well in a 96-well plate. The culture medium was supplemented with CaCl_2_ (final concentrations of 2 mM and 3 mM, in addition to the DMEM/F-12 medium formulation of ∼1mM calcium concentration) and *Lr*- derived EVs, followed by overnight incubation. To measure the intracellular calcium levels, Fluo-8 dye (Abcam) was prepared according to the manufacturer’s instructions. After overnight incubation, the Fluo-8 dye solution was added to each well and incubated for 30 min at 37°C, followed by an additional 30-min incubation at room temperature. Fluorescence intensity was measured using a Cytation 5 Cell Imaging Multimode Reader (BioTek) at excitation and emission wavelengths of 490/525 nm.

#### 3D spheroid culture conditions and staining

For spheroid formation, round-bottom-ultra-low attachment 96-well plates (Corning®, NY, USA) were used under sterile conditions. Cells were left in 2D culture until they reached 80% confluency, washed with 1X PBS, trypsinized with 0.25% Trypsin-EDTA (Thermo Fisher Scientific, MA, USA), and seeded at 5,000 cells per well in a 96 well plate in 100 μL of DMEM/F12 medium (complete medium). On Day 0, the plate was centrifuged at 0.147 rcf for 5 min and placed in an incubator overnight at 37°C under a 5% CO_2_ atmosphere. On Day 1, the cells were treated with DMEM/F12 media supplemented with CaCl_2_ at final concentration of 1.0 and 2.0 mM [Ca2+] and two concentrations of EVs (5 × 10^5^ EV per well; 5 × 10^8^ EV per well). Fresh EVs were added every 48 h for two weeks. Liposomes (L-alpha phosphatidylcholine and cholesterol control, Ø = 100 nm, Creative Biolabs, Inc., NY, USA) were used as the negative controls. Spheroid growth was assessed thrice per week using bright-field microscopy. After 14 days of culture, spheroids were fixed with 70% ethanol. Eight to ten spheroids per treatment were collected in 1.5 mL centrifuge tubes and fixed with 70% ethanol. The fixed spheroids were then sent to Histoserv, Inc. (Germantown, MD, USA) for paraffin embedding, microtome sectioning, and slide mounting. Each slide was sectioned at a thickness of 4 μm, deparaffinized, rehydrated, washed, and stained with hematoxylin and eosin, Alizarin Red, or von Kossa/Nuclear Fast Red using standard protocols. For von Kossa staining, 200 μL of a silver nitrate 5% solution was applied to each well for 15 min under direct light, followed by three washes with Milli-Q water. The spheroids were then treated with a sodium thiosulfate 5% solution for 5 min, washed twice with MilliQ water and counterstained with Nuclear Fast Red 0.1% solution (Sigma-Aldrich, MO, USA). For Alizarin Red staining, 200 μL of 40 mM ARS solution, pH 4.1 (Thermo Fisher Scientific, MA, USA) was added to each well and incubated at room temperature for 2 min. The dye was aspirated and each well was gently washed with Milli-Q water to remove non-specific binding. Plates were air-dried, spheroids were mounted on glass microscope slides, and examined under a bright-light microscope.

#### Biopsy tissue processing

Formalin-fixed paraffin-embedded (FFPE) tissue sections of eight high-grade DCIS lesions with comedonecrosis or solid features were used to extract proteins and DNA. Tissue sections were deparaffinized and stained with hematoxylin and eosin,^57^ then either scraped from the slide or subjected to laser capture microdissection (LCM)^58^ before extraction.^57^ LCM was performed using AccuLift Spatial Biology Profiler (Targeted Biosciences CAT: 10150/51, Figure S7). The workflow for this instrument included an initial whole-slide scan of the tissue slide utilizing AccuViz (Targeted Biosciences CAT: 12036) which acts as a liquid coverslip to improve the visualization of the tissue. This whole-slide scan was then uploaded to the cloud-based system where it was annotated by an expert pathologist to mark the cancerous regions of the DCIS slides. Next, the annotations were downloaded onto the AccuLift instrument and a slide dehydrated in xylene for 5 min was placed into the device. The same slide that was scanned and annotated was then microdissected autonomously using Acculift LCM caps (Targeted Biosciences CAT: 10004B). The microdissected tissue was then used for downstream analysis by applying 20 μL of lysis buffer directly to the surface of the Acculift LCM caps to elute all the captured material. The cytological and histological architecture of human breast cancer DCIS was unaltered before and after AccuLift microdissection. Both DNA and protein were extracted with high yield in comparison to the control scrape of the entire tissue slide.

#### Nucleic acid isolation and amplification

DNA from the scraped tissue slides and from LCM caps was isolated using phenol-chloroform-isoamyl alcohol in a ratio 25:24:1 and precipitated with 2.5M ammonium acetate and 2.5V of ethanol overnight at −20°C. The DNA was washed with 70% ethanol in deionized water, resuspended in TE buffer, and 100 ng was used for 16S rDNA PCR using Q-5 DNA polymerase. The following primers were used for PCR: bacterial 16S rDNA forward primer, 5′-TCCTACGGGAGGCAGCAGT-3′ reverse primer, 5′-GGACTACCAGGGTATCTAATCCTGTT-3′; *L*. *rhamnosus*-specific 16S rDNA forward primer, 5′-GGCGTGCCTAATACATGCAA-3′ reverse primer, 5′-GTCCGCCACTCGTTCAAAA-3′ and the amplicons were visualized by agarose gel electrophoresis^59^^,^^60^ Expressions of PMCA2, BCL-2, ORAI-1, and STIM1 in spheroids were analyzed using quantitative reverse transcription PCR. Five to seven spheroids were collected and centrifuged at 7000 × g for 10 min at room temperature. Spheroids were lysed, and RNA was isolated using TRIzol™ reagent (Thermo Fisher Scientific, MA, USA) following the manufacturer’s protocol. An iScript cDNA kit (Bio-Rad Laboratories) was used to reverse transcribe the RNA samples to cDNA, and quantitative RT-PCR was performed using Universal SYBR® Green Supermix (Bio-Rad Laboratories, CA, USA) on a QuantStudio™ 7 Pro Real-Time PCR system (Applied Biosystems, MA, USA). GAPDH was used as the housekeeping gene and the results were normalized to GAPDH ±standard deviation. Primers used are listed in Table S6.

### Quantification and statistical analysis

All results are expressed as mean +/− the standard deviation. For the proteomics experiments, the heatmap analysis was performed using Perseus software (Max Planck Institute of Biochemistry, Germany), and the Venn diagram was obtained using InteractiVenn (http://www.interactivenn.net/). A Wilcoxon rank-sum test was used to compare the abundance bacterial peptides grouped at the genus level in breast cancer cases and controls (*n* = 150 observations). A Chi square test was used to compare the frequency of bacterial peptides grouped at the species level in cases and controls (*n* = 150 observations). The circular taxonomic trees were created using the Interactive Tree of Life (iTOL, https://itol.embl.de). A Welch two sample t test was used to compare the intracellular calcium intensity of BT474 cells exposed to *Lactobacillus rhamnosus* EVs and negative controls using Fluo-8 staining (*n* = 7 observations). A Welch two sample t test was used to compare the calcium deposit staining abundance and gene expression abundance in BT474 spheroids treated with *Lr* extracellular vesicles and with negative controls (*n* = 7 observations). Analysis and plots were obtained using the R statistical software (www.r-project.org) and ImageJ (National Institutes of Health, USA). All replicates are independent biological replicates.

## Supplemental information

Document S1. Figures S1–S8 and Tables S4–S6

Table S1. Plasma proteomic data, related to Figure 1

Table S2. Serum proteomic data, related to Figure 1

Table S3. Saliva proteomic data, related to Figure 1

Data S1. Phylogenetic tree structure of bacterial species in plasma, related to Figure 2

Data S2. Phylogenetic tree structure of bacterial species in serum, related to Figure 2

Data S3. Phylogenetic tree structure of bacterial species in saliva, related to Figure 2

Data S4. Original images of western blots and micrographs related to Figures 4, 6 and 7
