# Supplementary material for: Lactobacillus rhamnosus-derived extracellular vesicles influence calcium deposition in a model of breast cancer intraductal calcium stress
Source: iScience. 2025 Apr 28;28(6):112538. doi: 10.1016/j.isci.2025.112538 (PMC12148604; doi:10.1016/j.isci.2025.112538)
Supplement: Document S1. Figures S1–S8 and Tables S4–S6 [file mmc1.pdf]

iScience, Volume *28*

Supplemental information

Lactobacillus rhamnosus-derived extracellular vesicles influence calcium deposition in a model of breast cancer intraductal calcium stress

Ngoc Vuong, Melany Alomia, Ahana Byne, Purva Gade, Thomas Raymond Philipson, Rayan Ibrahim Alhammad, Cade J. Skislak, Intisar Alruwaili, Fahad M. Alsaab, Weidong Zhou, Marissa Howard, Andrea Brothers, Amanda Haymond Still, Robyn P. Araujo, Monique Van Hoek, Barbara Birkaya, Virginia Espina, Richard A. Hoefer, Lance Liotta, and Alessandra Luchini

Supplementary Figures

A) *Bacillus filamentosus*

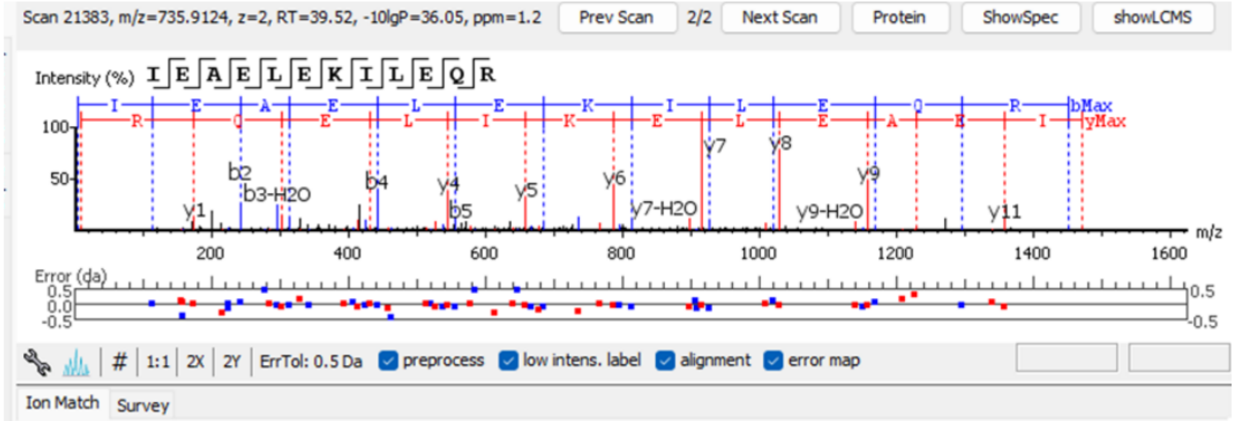

#	b	b-H2O	b-NH3	b (2+)	Seq	y	y-H2O	y-NH3	y (2+)	#
1	114.08	96.08	97.06	57.55	I					12
2	243.07	225.09	226.27	122.07	E	1357.84	1339.72	1340.62	679.60	11
3	314.23	296.24	297.14	158.01	A	1228.36	1210.50	1211.66	615.15	10
4	443.31	425.30	426.19	222.11	E	1157.70	1139.70	1140.63	579.33	9
5	556.40	538.43	539.27	278.18	L	1028.71	1010.57	1011.58	514.80	8
6	685.47	667.47	668.31	343.22	E	915.59	897.66	898.50	458.43	7
7	813.58	795.49	796.41	407.15	K	786.54	768.49	769.46	393.70	6
8	926.68	908.40	909.65	464.23	I	658.49	640.41	641.36	329.54	5
9	1039.60	1021.48	1022.58	520.25	L	545.37	527.41	528.39	273.15	4
10	1168.57	1150.76	1151.62	584.37	E	432.23	414.21	415.34	216.96	3
11	1296.77	1278.69	1279.68	648.37	Q	303.28	285.14	286.20	152.09	2
12					R	175.15	156.97	158.01	88.06	1

Figure S1. Example MS/MS spectrum of a microbiome-derived peptides detected in plasma, serum and saliva samples.

B) *Pseudomonas aeruginosa*

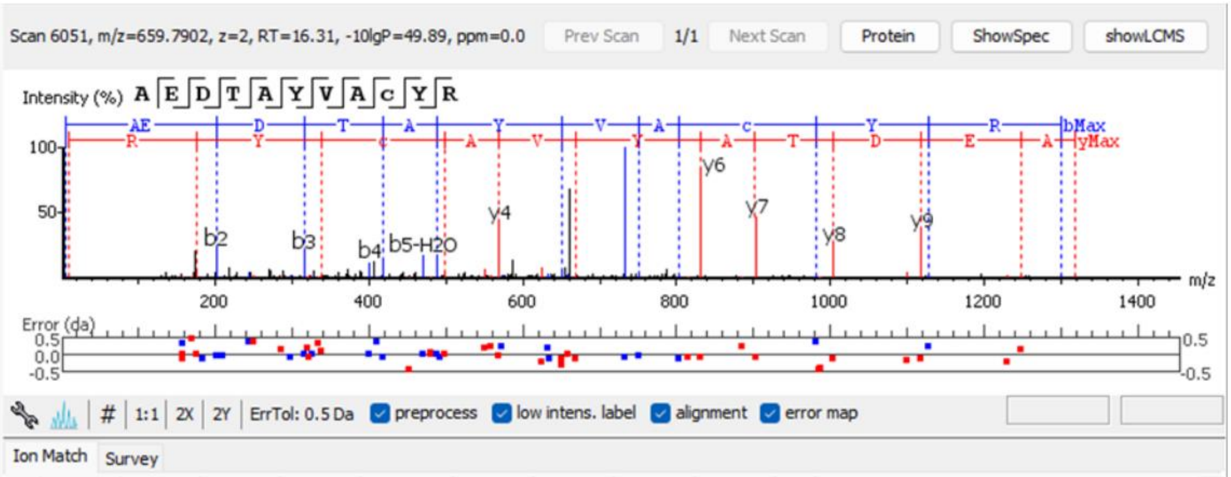

#	b	b-H2O	b-NH3	b (2+)	Seq	y	y-H2O	y-NH3	y (2+)	#
1	72.04	54.03	55.02	36.52	A					11
2	201.13	183.24	184.06	101.04	E	1247.40	1229.76	1230.51	624.52	10
3	316.13	298.21	299.09	158.23	D	1118.64	1100.69	1101.47	559.50	9
4	417.27	399.15	400.14	209.13	T	1003.63	985.94	986.85	502.23	8
5	488.21	470.21	471.17	244.20	A	902.53	884.41	885.12	452.20	7
6	651.44	633.05	634.38	326.10	Y	831.49	813.37	814.45	416.19	6
7	750.41	732.43	733.30	375.67	V	668.50	650.65	651.44	334.30	5
8	821.37	803.52	804.34	410.80	A	569.30	551.03	552.22	284.96	4
9	981.00	963.39	964.37	491.33	C(+57.02)	498.21	480.20	481.11	249.20	3
10	1144.46	1126.45	1127.17	572.48	Y	338.09	319.96	321.25	169.12	2
11					R	175.14	157.10	158.23	88.06	1

Figure S2. Example MS/MS spectrum of a microbiome-derived peptides detected in plasma, serum and saliva samples.

C) *Lactobacillus rhamnosus*

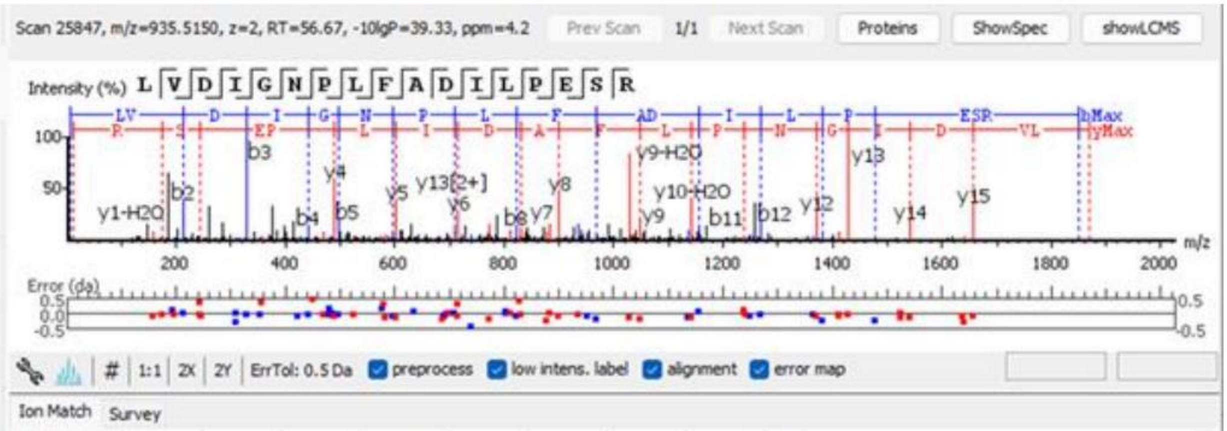

#	b	b-H2O	b-NH3	b (2+)	Seq	y	y-H2O	y-NH3	y (2+)	#
1	114.09	96.08	97.06	57.55	L					17
2	213.18	195.01	196.19	107.08	V	1755.93	1737.92	1738.90	878.72	16
3	328.26	310.49	311.18	164.59	D	1657.01	1638.97	1640.18	828.48	15
4	441.33	423.38	424.24	221.14	I	1542.02	1524.01	1524.82	771.62	14
5	498.35	480.36	481.11	249.65	G	1428.84	1410.81	1411.83	714.53	13
6	612.34	594.33	595.45	306.67	N	1371.84	1353.72	1354.70	686.59	12
7	709.38	691.51	692.44	355.26	P	1257.68	1239.52	1240.72	629.34	11
8	822.59	804.41	805.45	411.74	L	1160.63	1142.78	1143.60	580.48	10
9	969.76	951.67	952.51	485.29	F	1047.77	1029.70	1030.52	524.33	9
10	1040.58	1022.57	1023.55	520.79	A	900.62	882.50	883.45	450.29	8
11	1155.51	1137.76	1138.58	578.13	D	829.50	811.44	812.41	415.22	7
12	1268.74	1250.78	1251.66	634.78	I	714.53	696.40	697.42	357.32	6
13	1382.03	1363.86	1364.75	691.51	L	601.49	583.49	584.30	301.17	5
14	1479.12	1460.82	1461.80	740.38	P	488.38	470.33	471.22	244.24	4
15	1607.87	1589.86	1590.84	804.41	E	391.19	373.18	374.17	196.19	3
16	1694.90	1676.89	1677.87	847.95	S	262.15	244.24	245.25	131.58	2
17					R	175.19	157.21	158.09	88.06	1

Figure S3 Example MS/MS spectrum of a microbiome-derived peptides detected in plasma, serum and saliva samples.

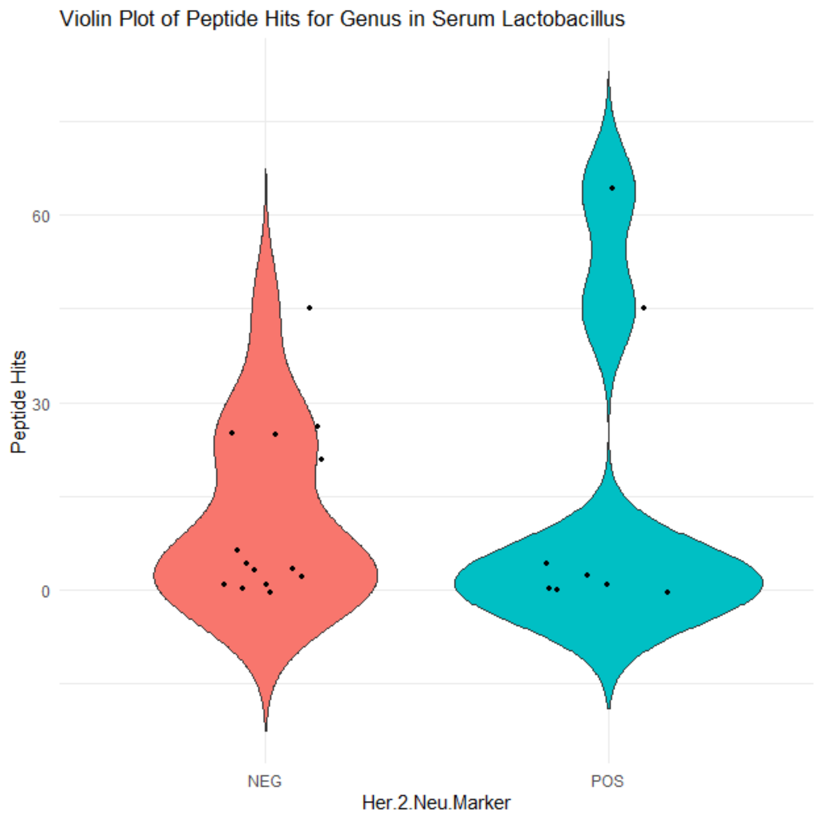

Figure S4. Correlation between *Lactobacillus* and clinical variables. Lactobacillus peptide hits were higher in Her2+ (mean=14.5) than Her2- (mean = 11.6) breast cancer patients**.**

PARTICLEMETRIX
www.particle-metrix.de

Electrophoresis & Brownian Motion
Video Analysis
Laser Scattering Microscopy

Operator (Report): pgade2
Video Operator: pgade2

Sample Parameters
Sample Name: 100Klacto2
Comment: Sample Remarks0:
Sample Remarks1:
Sample Remarks2:
Electrolyte:
Temperature: 22.46 °C sensed
pH 7.0 entered
Conductivity: 122.05 µS/cm sensed

Instrument Parameters
Laser Wavelength: 520 nm
Filter Wavelength: Scatter

Measurement Parameters
Cell S/N: Default

Measurement Mode: Size Distribution 3 Cycles
11 Positions, 1 Removed for Analysis

Result (sizes in nm)

	Number	Concentration	Volume
Median (X50)	99.5	99.5	157.3
Span	43.4	43.4	179.8

Concentration: 5.3E+7 Particles / mL
Dilution Factor: 1000
Original Concentration: 5.3E+10 Particles / mL

Quality
Average Counted Particles per Frame: 317
Number of Traced Particles: 4120

Analysis Parameters
Max Area: 1000, Min Area: 10, Min Brightness: 20

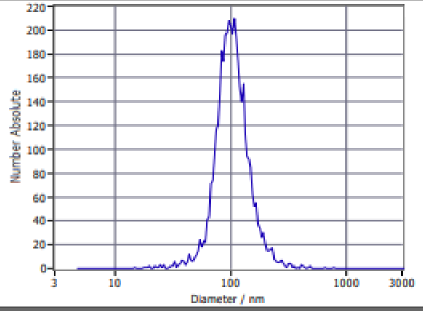

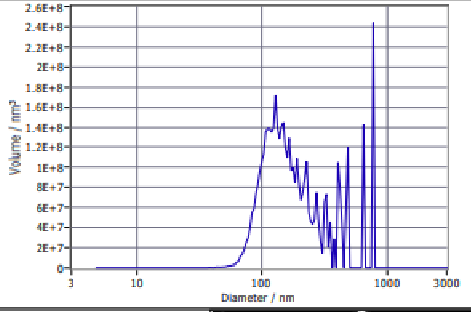

Peak Analysis (Concentration)

Diameter / nm	Number Absolute	FWHM / nm	Percentage
100.5	2.0E+2	64.0	100.0

X Values (all sizes are given in nm)

	Number	Concentration	Volume
X10	68.8	68.8	94.1
X50	99.5	99.5	157.3
X90	150.7	150.7	473.4
Span	0.8	0.8	2.4
Mean	108.8	108.8	232.7
StdDev	43.4	43.4	179.8

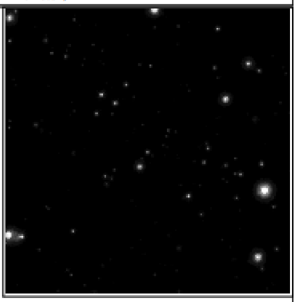

Comment

(Signature)

Figure S5. The ZetaView Particle Metrix analysis of *Lactobacillus rhamnosus*-derived EVs provides the number and size distribution. These data are relative to EVs collected from *L. rhamnosus* cultures allowed to grow for 4.5 hours.

Electrophoresis & Brownian Motion
Video Analysis
Laser Scattering Microscopy

PARTICLE METRIX
www.particle-metrix.de

Operator (Report): pgade2
Video Operator: pgade2

Sample Parameters
Sample Name: 100Klacto
Comment: Sample Remarks0:
Sample Remarks1:
Sample Remarks2:
Electrolyte:
Temperature: 22.65 °C sensed
pH 7.0 entered
Conductivity: 13.14 µS/cm sensed

Instrument Parameters
Laser Wavelength: 520 nm
Filter Wavelength: Scatter

Measurement Parameters
Cell S/N: Default

Measurement Mode: Size Distribution 3 Cycles
11 Positions, 1 Removed for Analysis

Result (sizes in nm)

	Number	Concentration	Volume
Median (X50)	111.5	111.5	294.0
Span	70.2	70.2	179.9

Concentration: 2.5E+7 Particles / mL
Dilution Factor: 50000
Original Concentration: 1.3E+12 Particles / mL

Quality
Average Counted Particles per Frame: 151
Number of Traced Particles: 2262

Analysis Parameters
Max Area: 1000, Min Area: 10, Min Brightness: 20

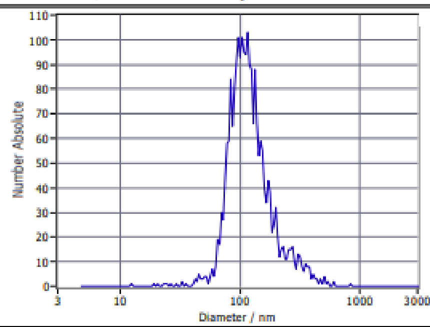

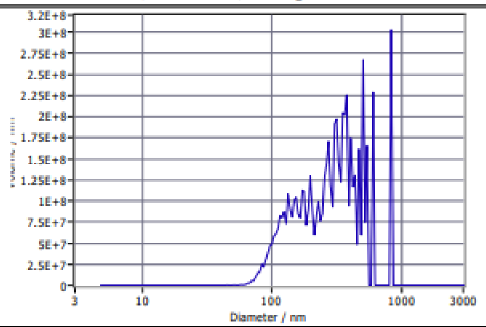

Peak Analysis (Concentration)

Diameter / nm	Number Absolute	FWHM / nm	Percentage
261.6	1.5E+1	78.3	100.0

X Values (all sizes are given in nm)

	Number	Concentration	Volume
X10	76.1	76.1	119.3
X50	111.5	111.5	294.0
X90	202.9	202.9	530.6
Span	1.1	1.1	1.4
Mean	132.6	132.6	321.6
StdDev	70.2	70.2	179.9

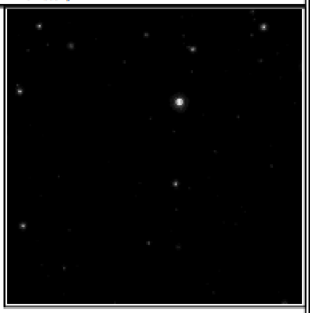

Comment

(Signature)

Figure S6. The ZetaView Particle Metrix analysis of *Lactobacillus rhamnosus*-derived EVs provides the number and size distribution. These data are relative to EVs collected from *L. rhamnosus* cultures allowed to grow for 24 hours.

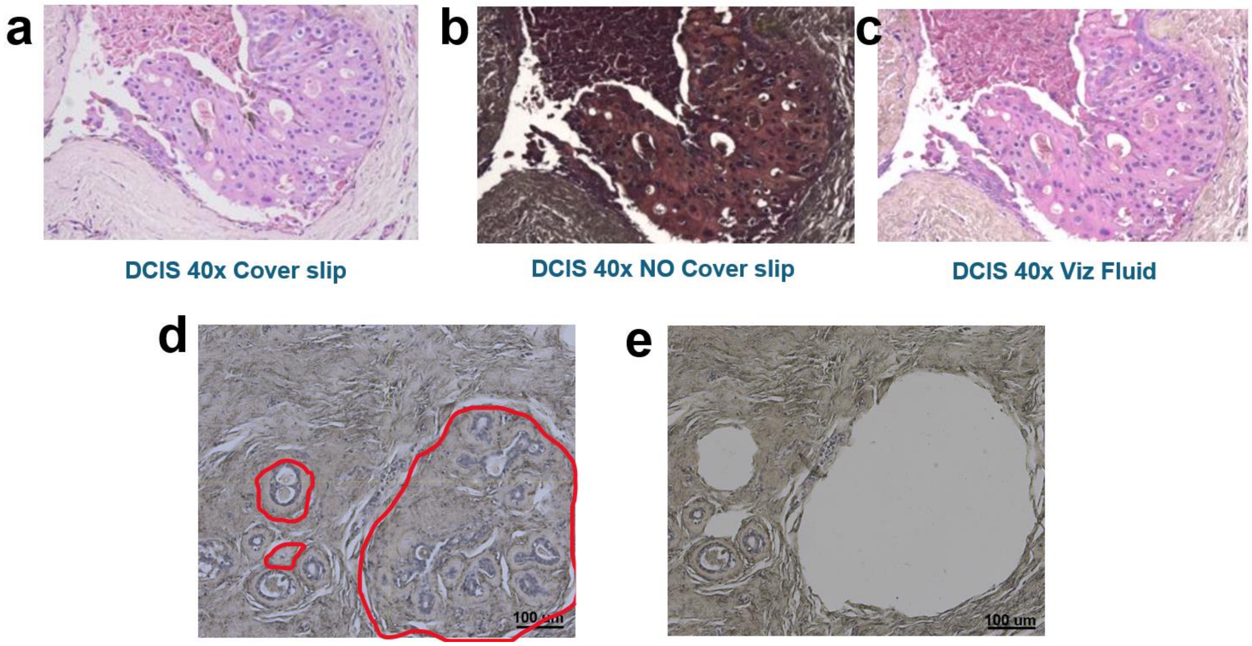

Figure S7. The laser capture microdissection workflow isolates intraductal tissues for protein and DNA extraction. Hematoxylin and eosin staining of human breast tissue shows pathological findings commonly observed in patients with ductal carcinoma in situ (DCIS) lesions. Panels **a-c** illustrate how consumables in the Acculift LCM Reagent Kits (Targeted Biosciences) improve image quality (cover slip, **a,** and visualization fluid, **b**) compared with the absence of consumables (**c**)**.** Panels **d** and **e** illustrate the results of laser capture microdissection, where the intraductal tissue is highlighted (**d**) and subsequently removed (**e**) for precision molecular analysis. The Targeted Biosciences AccuLift Spatial Biology Profiler was used for autonomous LCM through remote annotation of full-scanned slides.

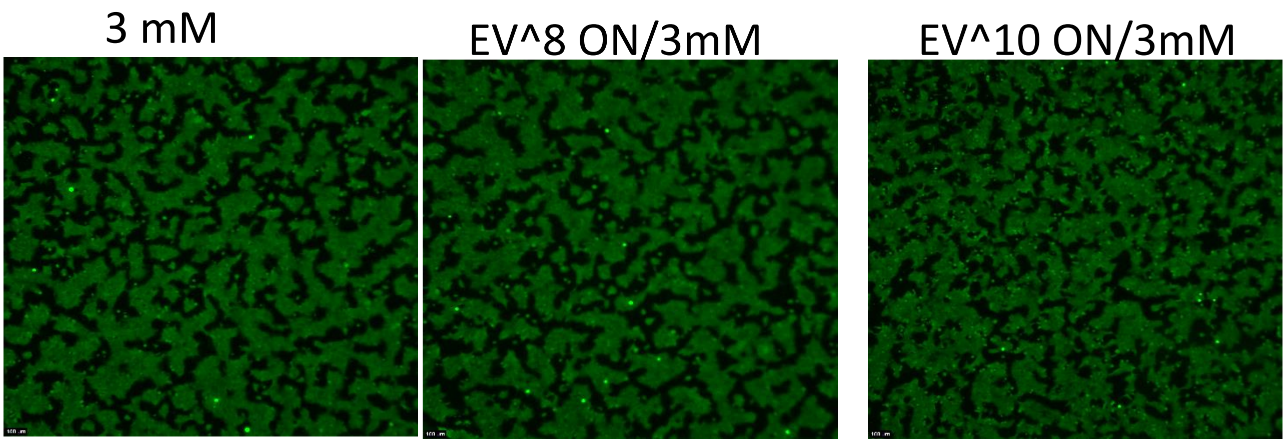

Figure S8. Fluorescence Microscope (Leica Thunder Imager DMi8, resolution XY = 2.7 µm, Z = 38 µm) images of BT-474 human breast cancer cells incubated with Fluo8 intracellular calcium stain. Reported conditions include: 3 mM supplemented calcium and no EVs = 3 mM, 3 mM supplemented calcium and 10^8 EVs = EV^8 ON/3mM, and 3 mM supplemented calcium and 10^10 EVs = EV^10 ON/3mM.

Supplementary Tables

Table S4: List of proteins identified in *L. rhamnosus* EVs and their biological functions.

Protein description	Protein ID (Uniprot)	Biological function
Enolase<br>Diphosphomevalonate decarboxylase<br>Phosphoenolpyruvate-protein phosphotransferase<br>Phosphoglycerate kinase<br>Glucokinase	C2JVV5<br>C2JXB8<br>C2JY89<br>C2JVV3<br>C2JXX8	Example metabolic enzymes of the glycolysis, phosphate pentose, hexosamine biosynthesis, and mevalonate pathways
Chaperone protein DNAK	C2JXM9	Protein folding
D-alanine--D-alanine carrier protein ligase	C2K1N0	Lipoteichoic acid biosynthesis
DD-transpeptidase<br>Glycosyl hydrolase family 25<br>Phosphoglucosamine mutase	C2K1P6<br>C2JVK8<br>C2JW02	Peptidoglycan biosynthesis/remodeling
Capsular polysaccharide biosynthesis protein CpsC<br>Tyrosine-protein kinase CpsD	C2JZY3<br>C2JZY4	Capsular polysaccharides synthesis
PIII-type proteinase	C2JZ55	Cell-envelope proteinase involved in β-casein proteolysis
Putative ribosomal protein S1<br>Exonuclease<br>Elongation factor P<br>DNA ligase<br>DNA polymerase I<br>Cysteine--tRNA ligase	C2JWW5<br>C2K0Y0<br>C2JXW1<br>C2JW40<br>C2JY30<br>C2JZ02	DNA and RNA metabolism

Table S5: List of bacteria used to generate the microbiome database from the NCBI protein database repository.

Veillonella dispar	*Bacteroides spp.*
Fusobacterium spp.	*Parabacteroides distasonis*
Megamonas spp.	*Lactobacillus spp.*
Prevotella copri	*Collinsella aerofaciens*
Porphyromonas gingivalis	*Pseudomonas aeruginosa*
Escherichia coli	*Bacillus spp.*
Aspergilla niger	*Corynebacteria urealyticum*
Corynebacterium group D2	*Bifidobacterium animalis*
Sphingomonas spp.	*Moraxella spp.*
Prevotella nigrescens	*Brevundimonas spp.*
Streptococcus lactis	*Ralstonia spp.*
Enhydrobacter spp.	*Gluconacetobacter spp.*
Proteus spp.	*Methylobacter spp.*
Ruminococcus spp.	*Anaerotruncus spp.*

Table S6: Primers used for real-time PCR assays

Gene name	Transcript ID	Forward primer (5'-3')	Reverse primer (5'-3')
PMCA2	ENST00000360273.7	TCCTCAACGAACTCACCTGC	GCCGTGTTGATATTGTCGCC
BCL-2	ENST00000333681.5	GGATAACGGAGGCTGGGATG	GGCCAAACTGAGCAGAGTCT
ORAI-1	ENST00000617316.2	GCTCTGCTGGGTCAAGTTCT	CGTTGAGCTCCTGGAACTGT
STIM-1	ENST00000616714.4	CCATCACCACTACCACCACC	CCAAGCTCTCTGAATGCCCA
GAPDH	ENST00000396861.5	GTTCGACAGTCAGCCGCATC	GGAATTTGCCATGGGTGGA
