# Supplementary material for: Lactobacillus rhamnosus-derived extracellular vesicles influence calcium deposition in a model of breast cancer intraductal calcium stress
Source: iScience. 2025 Apr 28;28(6):112538. doi: 10.1016/j.isci.2025.112538 (PMC12148604; doi:10.1016/j.isci.2025.112538)
Supplement: Data S4. Original images of western blots and micrographs related to Figures 4, 6 and 7 [file mmc8.pdf]

Data S4. Original images of western blots and micrographs related to Figures 4, 6 and 7.

Figure 4

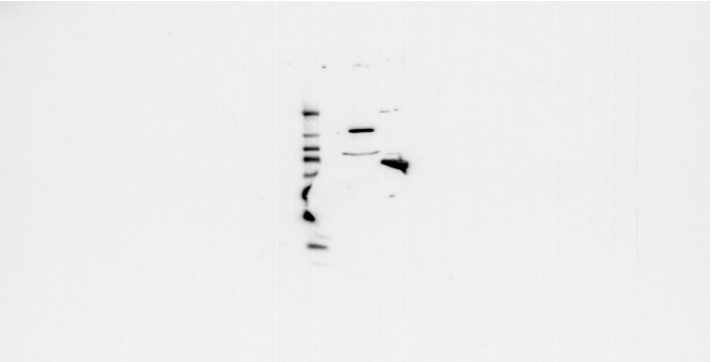

CD63

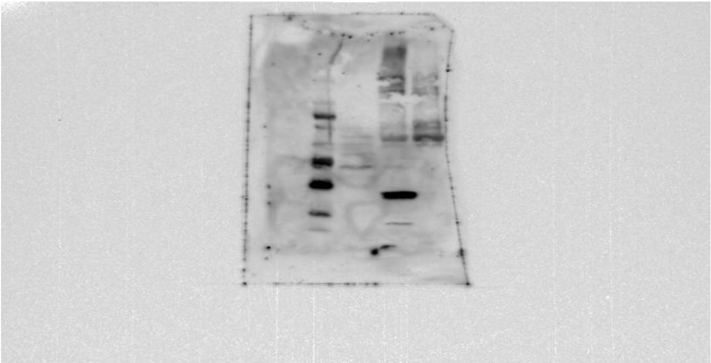

CD9

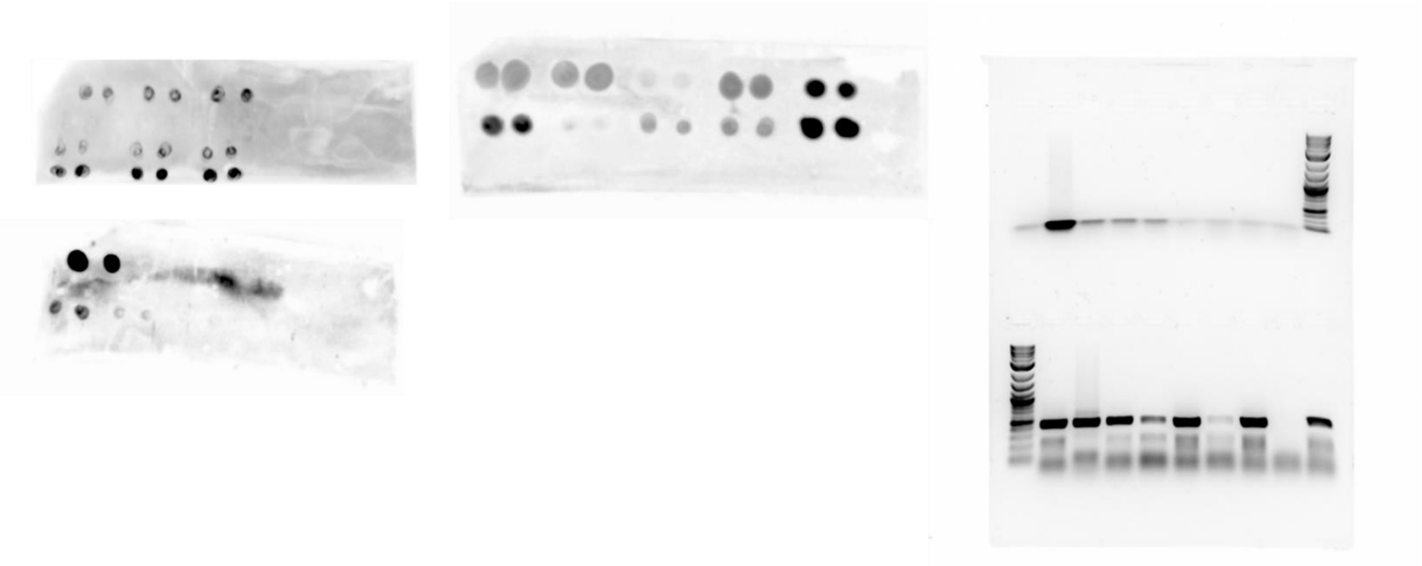

Figure 6

Day 1

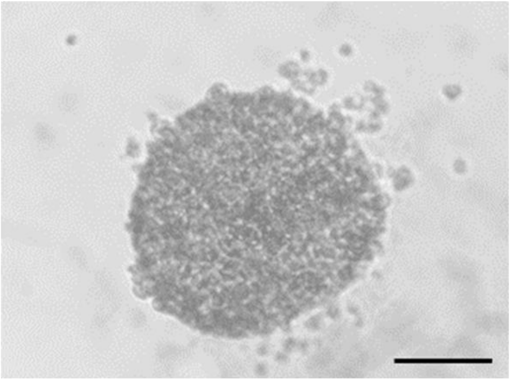

Day 14

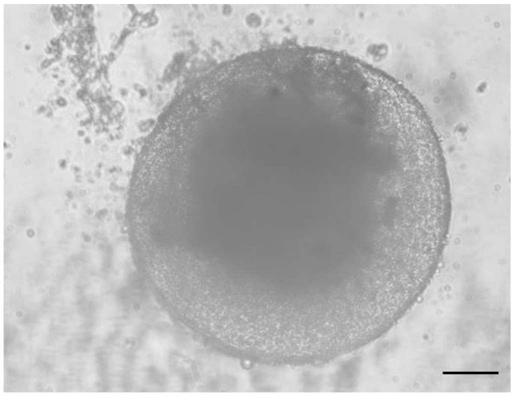

The bar corresponds to 200 μm

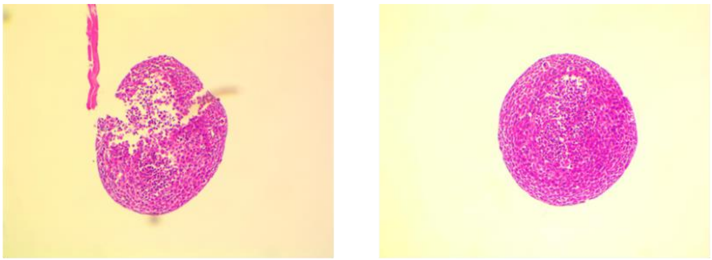

10X magnification

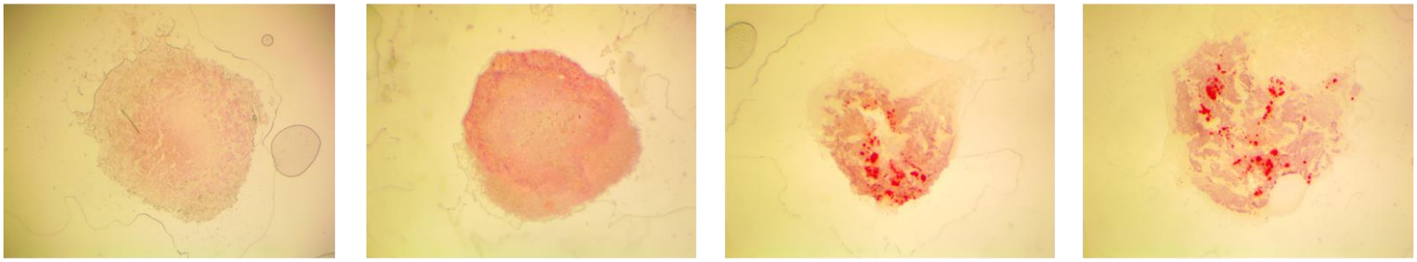

10X magnification

Figure 7A

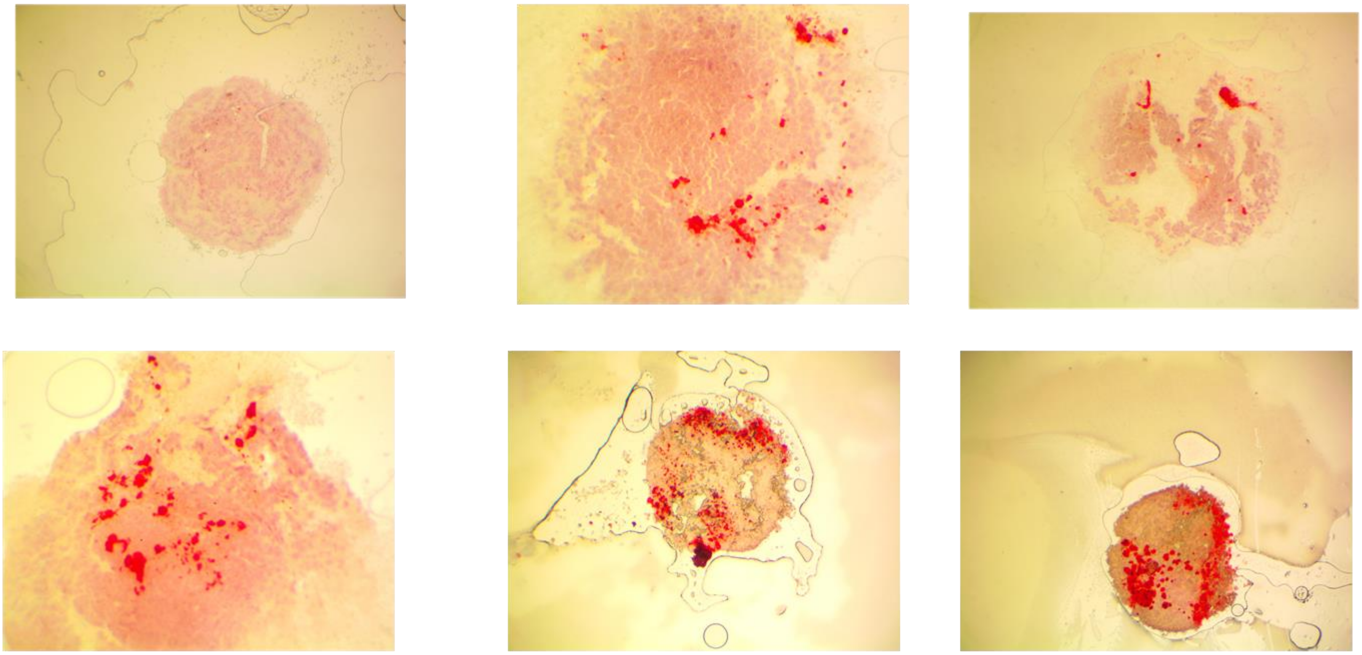

10X magnification

Figure 7C

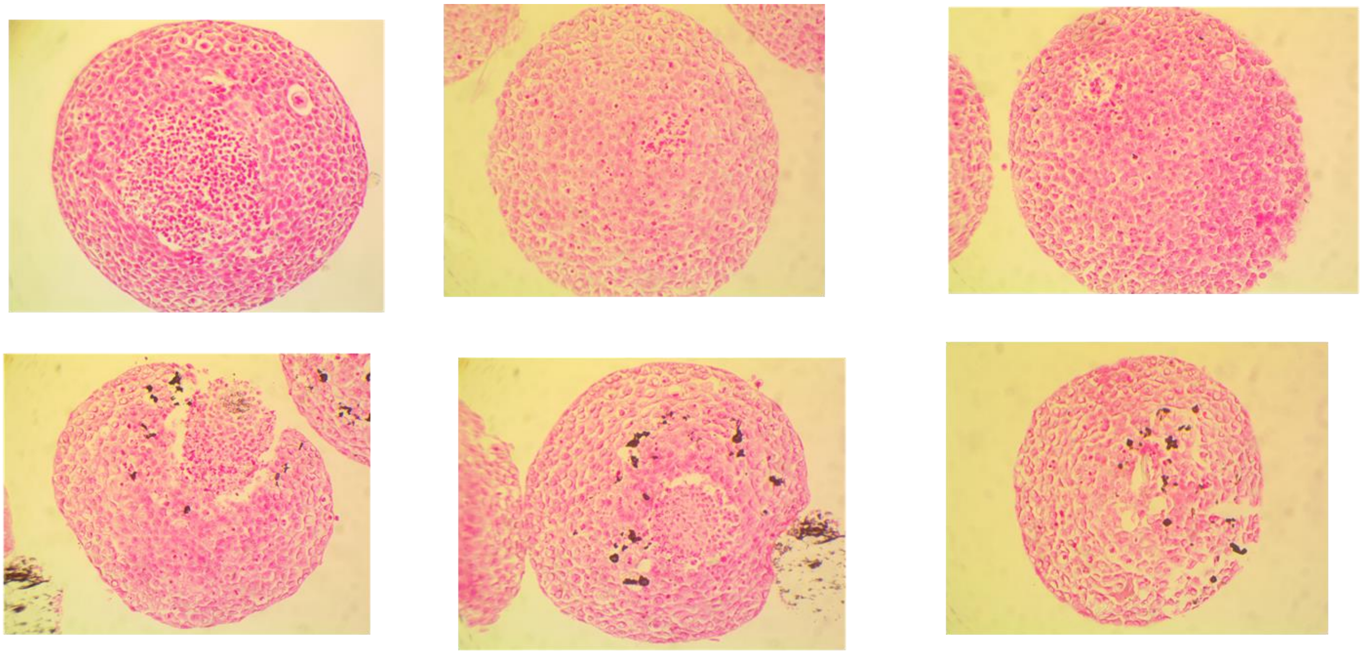

20X magnification

Figure 7E

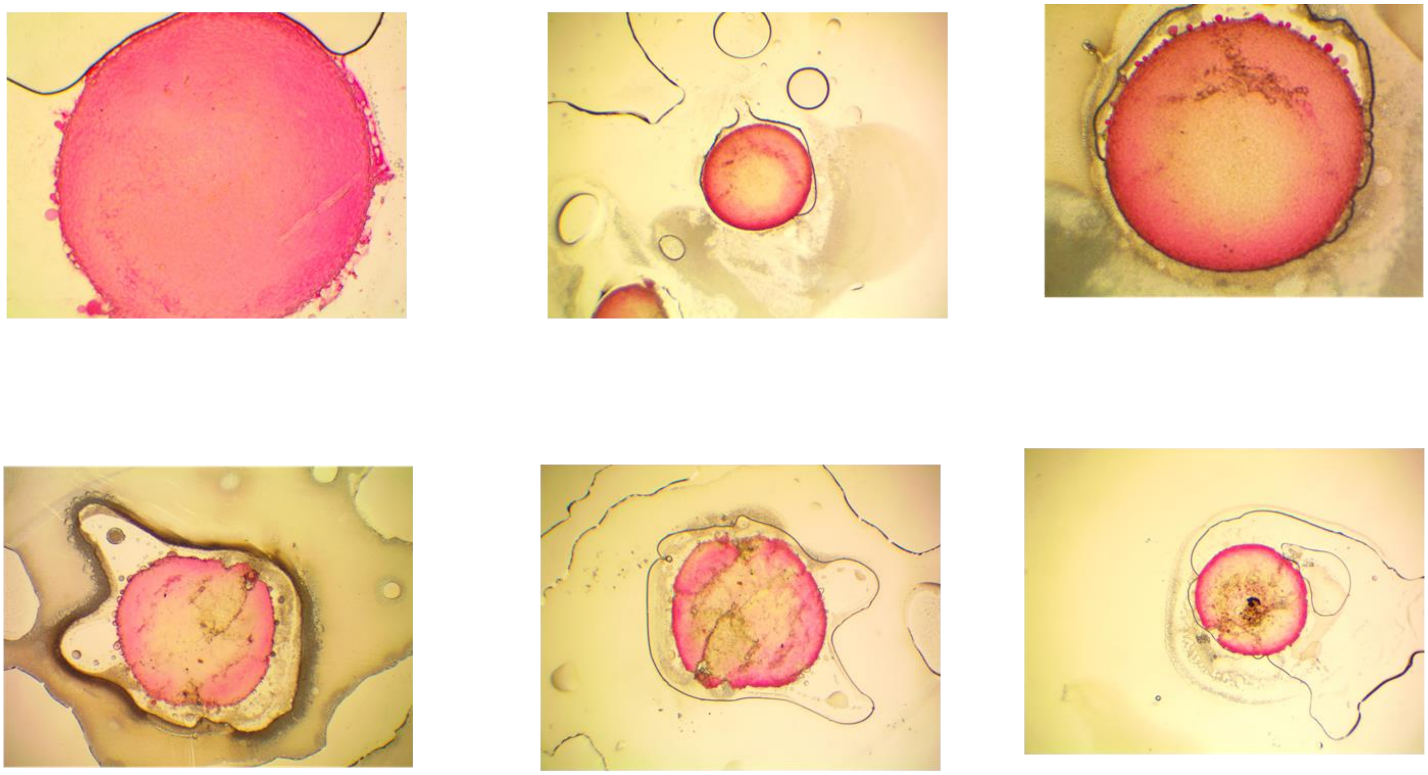

10X magnification
